# SkinNet-X: a task coupled feature interaction framework for joint skin lesion segmentation and classification

**DOI:** 10.3389/frai.2026.1886221

**Published:** 2026-09-30

**Authors:** E. Babu, S. Murali

**Affiliations:** School of Computer Science and Engineering (SCOPE), Vellore Institute of Technology, Vellore, Tamil Nadu, India

**Keywords:** cross task consistency learning, deep learning, dermoscopic image analysis, multitask learning, skin lesion analysis, skin lesion classification, skin lesion segmentation

## Abstract

Well trained segmentation and classification of skin lesions from dermoscopic images are crucial to an early detection of melanoma and other malignant skin disease. While many deep learning models reach impressive overall performance, most existing approaches treat segmentation and classification as separate tasks, leading to limited interaction between spatial lesion localization and semantic disease knowledge. This segregation results in a high variance prediction and would reduce the overall robustness of automated diagnostic systems. To mitigate this limitation, we proposed SkinNet-X: a unified multitask framework to reformulate the skin lesion segmentation and classification tasks jointly. We achieve this with a shared hierarchical feature representation to transfer knowledge between both tasks while introducing new Cross Task Consistency Learning (CTCL) strategy that enforces semantic consistency between lesion localization and disease prediction during joint optimization. In order to enhance the discriminative capability of feature expression and alleviate redundant representations, a dual stage multi label feature optimization module is further exploited, accompanied by an adaptive PolyLoss based optimization strategy stabilizing multitask learning. The effectiveness of the proposed SkinNet-X framework was evaluated using segmentation and classification experiments on the ISIC 2018, ISIC 2019, and HAM10000 benchmark datasets. We also performed additional analyses, namely ablation studies, computational complexity evaluation, sensitivity analysis and statistical validation to explore the contribution of each architectural component. Experimental results show that the proposed multitask learning strategy can consistently improve accuracy of lesion localization and performance of classification with robust generalizability across multiple datasets. These findings demonstrate that SkinNet-X is an effective and potentially useful framework for automated skin lesion analysis and computer-aided dermatological diagnosis.

## 1 Introduction

Skin cancer is one of the most prevalent developing malignancies in the world, and represents considerable public health burden. Recent worldwide cancer data reveal that melanoma has the poorest prognosis among skin cancers due to its strong capacity to spread and high fatality rate, especially when identified at late stages. The importance of CAD systems that can consistently support dermatologists in their normal clinical examination is highlighted by clinical research that consistently demonstrates that the earlier a disease is recognized, the greater the patient survival rate. Due to its ability to visualize small morphological features that are undetectable to the unaided eye, dermoscopic imaging has become the gold standard non-invasive clinical imaging method for assessing skin lesions.

However, because lesion color, texture, size, form, illuminance, and border irregularity vary greatly, automatic interpretation of dermoscopic pictures is a highly difficult computer vision task. The last 10 years have seen tremendous progress in automated skin lesion diagnosis thanks to deep learning discoveries that enable end-to-end learning of discriminative picture representations. While classification techniques employ global semantic context to predict lesion classifications, segmentation techniques are primarily used to reveal the exact borders of lesions. Even though there is a substantial clinical correlation between the two tasks, geographical localization and semantic recognition are usually adjusted independently with little complementary information transfer. Therefore, while classification models may fail to localize a significant aspect of morphology if explicit spatial localization cannot be effectively incorporated into the learned features, segmentation models can accurately delineate anatomical lesion boundaries without always including sufficient disease specific features. Multitask learning has become a potential paradigm for concurrently doing out lesion segmentation and classification inside a single framework in order to get beyond these restrictions. Multitask learning can increase computing efficiency and facilitate knowledge transfer between location and recognition by sharing feature representations across several related tasks. However, the majority of multitask frameworks now in use rely on straightforward feature fusion or parameter sharing procedures, implicitly assuming that the acquired representations are inherently consistent across tasks. In reality, distinct feature distributions are frequently optimized by segmentation and classification targets, which cause task inconsistency during training. These discrepancies might restrict diagnostic performance by decreasing feature discriminability and weakening optimization stability.

Attention mechanisms, feature fusion techniques, supplementary supervision, or consistency requirements have all been used in a number of recent research to improve multitask learning. However, rather than directly modeling semantic agreement between lesion location and illness prediction, these methods often concentrate on enhancing feature aggregation. Because of this, there is little constraint on the link between segmentation and classification, which permits both tasks to converge toward distinct optimization goals. Furthermore, by combining many pretrained networks or optimization modules, many current frameworks highlight architectural complexity without offering a sound method that guarantees cross task semantic consistency during the learning process.

Inspired by these findings, this study presents SkinNet-X, a unified multitask framework that uses a Cross Task Consistency Learning (CTCL) approach to explicitly build semantic connection between lesion segmentation and illness categorization. The proposed CTCL mechanism enables segmentation and classification branches to generate mutually consistent semantic representations during joint optimization, in contrast to traditional multitask learning techniques that just share intermediate features. The developed approach increases feature discriminability, boosts optimization stability, and encourages more accurate lesion localization and illness identification by lowering task inconsistency. Additionally, the system uses a joint optimization technique that combines segmentation supervision, classification supervision, and cross task consistency regularization into a single end-to-end learning framework, as well as a common hierarchical feature representation. Extensive experiments are conducted using three publicly accessible benchmark datasets: ISIC 2018 for lesion segmentation and ISIC 2019 and HAM10000 for disease classification.

The primary contributions of this work are summarized as follows:

1. To facilitate efficient knowledge transfer between the two clinical tasks, a unified multitask framework (SkinNet-X) is presented for simultaneous skin lesion segmentation and classification utilizing a common hierarchical feature representation.
2. To improve feature discriminability and optimization stability during multitask learning, a novel Cross Task Consistency Learning (CTCL) technique is presented that directly minimizes semantic inconsistency between lesion location and illness detection.
3. To enhance lesion border localization and diagnostic classification in a single end-to-end training procedure, a joint optimization framework including segmentation supervision, classification supervision, and cross task consistency regularization is created.
4. To show the efficacy and resilience of our framework, a thorough experimental validation is carried out using several benchmark datasets through quantitative evaluation, ablation analysis, computational complexity assessment, repeated-run statistical stability analysis, and qualitative visualization.
5. An early combination of adaptive feature optimization and cross task consistency learning for unified skin lesion segmentation and classification is shown in this study.

This is how the rest of the paper is structured. Related work is reviewed in Section 2. The proposed technique is explained in Section 3. The findings and discussion of the experiment are presented in Section 4. The article is concluded and next work is outlined in Section 5.

## 2 Related work

Recent advances in deep learning have significantly improved automated skin lesion analysis through encoder decoder segmentation networks and convolutional neural network (CNN) based classification frameworks. Early studies primarily employed sequential pipelines in which lesion segmentation was first performed to localize suspicious regions, followed by independent disease classification. Zafar et al. (2023) proposed a MobileNetV2 based DeepLabV3+ framework combined with DenseNet201 and Slime Mould Algorithm (SMA) based feature selection for multiclass skin lesion classification. Kumar et al. (2025) developed a hybrid framework integrating DeepLabV3+, Adaptive Contextual Gray Level Co-occurrence Matrix (GLCM), and an LSTM classifier to capture contextual texture information.

Similarly, Shahzaib et al. (2025) introduced an Efficient Hair Removal (EHR) framework employing Deep Residual UNet segmentation and DenseNet based classification to improve diagnostic performance through artifact removal. More recently,(Arshad et al. 2025) proposed an improved ResNet20 based DeepLabV3+ segmentation network together with MAKNet100 using network level feature fusion and Bayesian optimization for enhanced segmentation and recognition. Although these approaches demonstrated competitive performance, segmentation and classification were generally optimized sequentially, limiting effective knowledge sharing between lesion localization and disease prediction.

To overcome the limitations of sequential learning, multitask learning frameworks have recently attracted considerable attention by simultaneously optimizing lesion segmentation and classification within a unified architecture. Owida et al. (2025) proposed a dual branch framework that jointly learned segmentation and classification through shared feature representations, while Thapar et al. (2024) integrated the Grasshopper Optimization Algorithm with CNN based segmentation and melanoma classification to enhance feature selection and diagnostic accuracy. Rokade and Mishra (2024) further incorporated blockchain technology and Transit Circle Inspired Optimization (TCIO) to improve classification reliability and secure medical data management. These studies demonstrate the effectiveness of multitask optimization; however, most existing frameworks rely primarily on feature sharing or optimization strategies without explicitly enforcing semantic consistency between lesion localization and disease recognition during joint learning.

In parallel, substantial efforts have focused on improving skin lesion classification using advanced feature extraction and attention mechanisms. Aruk et al. (2025) proposed a hybrid ConvNeXt based architecture for capturing discriminative lesion representations, whereas Umapathi et al. (2025) optimized the Inception ResNet-V2 architecture to improve multiclass skin cancer classification. Hasan et al. (2026) introduced a Tri-Path Attention Stacked Ensemble architecture employing multiple EfficientNet based learners to strengthen feature discrimination. Hu et al. (2024) proposed multi scale feature fusion with class weighted loss and EfficientNetV2 to address severe class imbalance.

While Tran-Van and Le (2025) utilized cross attention fusion to integrate dermoscopic images with clinical metadata. Furthermore, Rajeshkumar and Chowdhary (2026) combined CNN and BiLSTM architectures for contextual feature learning, whereas Padhy et al. (2025) integrated ResNet50 with LSTM to enhance sequential representation learning. Although these methods substantially improve classification accuracy, they focus exclusively on disease recognition and do not exploit lesion localization information to guide semantic feature learning. Recent studies have also explored segmentation refinement, explainability, and foundation models to improve medical image analysis. Dzieniszewska et al. (2025) introduced deep pixel wise supervision to guide lesion aware feature learning during classification, while Yang et al. (2025) incorporated transfer learning, channel spatial attention, Gradient Reversal Layer (GRL), and GradCAM visualization to improve segmentation interpretability and domain adaptation.

Our previous work, AttenUNeTX Babu and Murali, (2025), employed iterative feedback refinement, attention mechanisms, and an Order Statistics Layer (OSL) to enhance lesion boundary delineation through progressive segmentation refinement. Similarly, Shaik et al. (2025), Efat (2026), Ghazouani (2025), Li et al. (2025), and Ruga et al. (2026) further improved skin lesion classification using attention based hybrid learning, feature enhancement, transformer interaction modules, residual attention, and explainable AI strategies; however, these methods mainly focused on classification and did not explicitly integrate segmentation guided semantic consistency.

More recently, Liu and Xu (2025) proposed HF-SAM, which adapts the Segment Anything Model (SAM) using Low Rank Adaptation (LoRA), Adaptive Weighted Feature Fusion Module (AWFFM), and a two step skip feature fusion decoder for few shot medical image segmentation. Although these approaches improve segmentation quality and feature representation, they remain primarily segmentation oriented and do not explicitly establish semantic interaction between lesion localization and disease classification.

However, Rahman et al. (2025) proposed an unsupervised skin lesion classification framework integrating fine-tuned ESRGAN-based image enhancement, histogram-based feature extraction, PCA and t-SNE dimensionality reduction, and K-means clustering. Their approach demonstrated that enhancement of dermoscopic image quality can improve the visibility of discriminative lesion characteristics before subsequent feature extraction and classification. The method achieved classification accuracies of 87.77% and 90.50% on ISIC2019 and HAM10000, respectively. However, the framework primarily addresses unsupervised lesion classification and does not explicitly model the semantic interaction between lesion segmentation and disease classification. In contrast, the proposed SkinNet-X jointly optimizes lesion localization and disease recognition and introduces CTCL to regularize cross-task semantic consistency during multitask learning.

Overall, existing studies mainly follow four research directions: (i) sequential segmentation, classification pipelines, (ii) multitask learning through feature sharing, (iii) classification oriented deep learning frameworks, and (iv) segmentation refinement and explainable learning strategies. Despite achieving remarkable performance, most existing methods either optimize segmentation and classification independently otherwise exchange information through simple feature sharing, attention fusion, or optimization strategies. Consequently, semantic inconsistency between lesion localization and disease recognition remains insufficiently addressed, potentially limiting optimization stability and feature discriminability. Motivated by these observations, the proposed **SkinNet-X** introduces a novel **Cross Task Consistency Learning (CTCL)** mechanism that explicitly enforces semantic agreement between segmentation and classification during end-to-end multitask optimization, thereby improving both lesion localization and disease prediction.

### 2.1 Research gap and motivation

Using convolutional neural networks, vision transformers, attention mechanisms, and foundation models, recent developments in deep learning have greatly enhanced the segmentation and classification of skin lesions. Jointly improving segmentation and classification can increase computing efficiency and encourage feature sharing between related tasks, as several multitask learning frameworks have shown. Additionally, consistency learning techniques such as teacher student learning, Kullback-Leibler divergence regularization, contrastive learning, and mutual information maximization have demonstrated encouraging results in semi-supervised learning and domain adaptation.

Three significant research gaps still exist in spite of these developments.

First, the majority of skin lesion analysis techniques now in use optimize segmentation and classification separately, making it impossible for spatial lesion localization and semantic illness detection to effectively interact. As a result, the clinically significant lesion morphology that is retrieved during segmentation is not properly utilized to enhance the categorization of diseases.

Second, shared encoders, feature fusion modules, or attention based interaction mechanisms are typically used in multitask learning frameworks. The semantic coherence between segmentation predictions and classification judgments during optimization is not explicitly constrained by these methods, despite the fact that they enhance information sharing across various tasks. Consequently, the efficiency of combined learning may be limited if both tasks eventually acquire inconsistent feature representations.

Third, the major goal of existing consistency learning strategies is to preserve prediction consistency within a single learning objective using methods like mutual information maximization, contrastive representation learning, and KL-divergence regularization. The semantic agreement between pixel level lesion localization and image level illness recognition, which reflect essentially separate learning aims, is not particularly modeled by these techniques.

SkinNet-X, a unified multitask framework with a revolutionary Cross Task Consistency Learning (CTCL) method, in response to these restrictions. The CTCL explicitly regularizes the interaction between the segmentation and classification branches by encouraging both tasks to learn mutually consistent semantic representations throughout the optimization process, in contrast to traditional multitask learning techniques that only rely on implicit feature sharing. As a result, the multitask framework delivers more accurate lesion localization and illness detection while also improving feature discriminability and optimization stability. A comparative synopsis of recent skin lesion research methods, datasets, performance measures, and limitations is presented in Table 1.

**Table 1:** Synopsis of skin lesion research methods, datasets, and performance measures.

References	Model/approach	Key technique	Dataset	Performance	Limitation
Owida et al. (2025)	Dual Branch Framework	EfficientNet + Transformer + ASPP	HAM10000, ISIC	Acc: 97.08%	High complexity, loose task coupling
Tran-Van and Le (2025)	Multimodal Cross Attention	Image + Metadata Fusion	HAM10000	Acc: 95.73%	Requires multimodal data, deployment complexity
Thapar et al. (2024)	Grasshopper Optimization + CNN	Optimization based segmentation	ISIC, PH2	Acc: 98.52%	Limited generalization, handcrafted features
Aruk et al. (2025)	ConvNeXt + Vision Transformer	Hybrid CNN–ViT	HAM10000, ISIC2019	Acc: 94.30%	No segmentation integration
Shaik et al. (2025)	CNN + BiLSTM + Attention	Spatial & temporal attention	ISIC	Acc: 92.73%	Sequential modeling overhead
Umapathi et al. (2025)	Optimized Inception ResNetV2	Hyperparameter tuning + SMOTE	ISIC2019	Acc: 97.65%	Limited robustness to artifacts
Li et al. (2025)	Transformer Feature Interaction	Feature enhancement + attention	Custom dataset	Acc: 92.65%	Limited dataset diversity
Efat (2026)	Tri Attention EfficientNet	Multi attention + ensemble	HAM10000	Acc: 94.57%	Complex ensemble weighting
Padhy et al. (2025)	ResNet + LSTM	Temporal feature modeling	ISIC, HAM10000	Acc: 95.72%	High computational cost
Ghazouani (2025)	MuRANet	Multi scale attention CNN	ISIC, PH2	F1: 90%	Limited explainability
Ruga et al. (2026)	MultiExCam	DL + ML + Explainability	ISIC, HAM10000	AUC: 98%	Complex hybrid pipeline
Shahzaib et al. (2025)	EHR + Residual U Net	Hair removal + segmentation	ISIC2019	Acc: 97.74%	Preprocessing dependent

## 3 Proposed methodology

### 3.1 Overall framework of SkinNet-X

Precise lesion localization and trustworthy disease recognition are necessary for early and accurate diagnosis of skin lesions. While recent deep learning techniques have shown impressive progress in skin lesion segmentation and classification, these tasks are typically optimized independently or connected only through implicit feature sharing, which frequently results in inconsistent feature representations, limiting the interaction between lesion localization and disease recognition during joint learning.

As a result, classification may ignore fine grained lesion morphology due to the ineffective integration of explicit spatial information into semantic feature learning, whereas segmentation may precisely define lesion borders without adequately supporting diagnostic classification. In order to overcome these constraints, this paper presents SkinNet-X, a unified multitask learning framework that uses the Cross Task Consistency Learning (CTCL) mechanism to explicitly enforce semantic interaction between the two tasks while concurrently performing skin lesion segmentation and disease classification through a shared hierarchical representation. The multitask framework introduces an explicit consistency regularization strategy that encourages lesion localization and disease recognition to learn mutually compatible semantic representations throughout the optimization process, in contrast to traditional multitask architectures that only rely on shared encoders or attention based feature fusion.

The framework consists of eight sequential components:

1. Adaptive image enhancement
2. Shared hierarchical feature representation
3. Lesion segmentation branch
4. Disease classification branch
5. Cross task consistency learning (CTCL)
6. Training time dual stage feature optimization
7. Joint optimization strategy
8. Post processing and final prediction

To increase lesion visibility and reduce typical imaging errors such hair obstruction, light fluctuation, and acquisition noise, dermoscopic pictures are first treated to adaptive image enhancement. A shared hierarchical feature encoder then processes these improved pictures to generate discriminative multi scale representations that can allow both illness detection and lesion location at the same time. After then, two task specific branches get the acquired hierarchical characteristics. While the disease classification branch learns discriminative semantic representations for lesion categorization, the lesion segmentation branch predicts precise lesion borders by combining small spatial features with global contextual information. Complementary information may be shared throughout the learning process since both branches come from the same feature hierarchy.

The Cross Task Consistency Learning (CTCL) module is a significant addition to the multitask architecture. Current multitask learning techniques often make the assumption that segmentation and classification can work together through feature sharing alone. However, both tasks naturally acquire distinct feature distributions during optimization since classification does image level semantic recognition while segmentation performs dense pixel level prediction. By promoting mutual consistency between the representations produced by both branches throughout end-to-end learning, the proposed CTCL expressly reduces this semantic disparity. As a result, the framework increases feature discriminability, lowers optimization conflicts, and boosts diagnostic reliability. A training time dual stage feature optimization technique is included to further enhance optimization stability. Global exploration and local exploitation are used to find useful feature representations and maximize model convergence during network training. The proposed optimization strategy helps the network choose more discriminative feature subsets while eliminating redundant representations, in contrast to traditional optimization techniques that only use gradient based learning. There is no extra computing burden during inference because this optimization is carried out only during training.

Finally, segmentation supervision, classification supervision, consistency regularization, and network regularization are collaboratively optimized using a unified objective function, enabling all network components to be trained simultaneously inside a single end-to-end framework. The improved network generates precise lesion segmentation and dependable illness classification during inference. Lightweight post processing is then used to increase prediction robustness and segmentation boundaries.

SkinNet-X offers three significant benefits over traditional multitask learning systems. First, effective information transfer between lesion location and illness recognition is made possible by the same hierarchical structure. Second, instead of depending only on implicit feature sharing, the proposed CTCL explicitly models semantic agreement between the two tasks. Third, the developed architecture is appropriate for realistic computer aided skin lesion analysis as the unified optimization framework improves convergence stability while preserving computational economy. The overall architecture of the proposed SkinNet X framework is illustrated in Figure 1.

**Figure 1:**
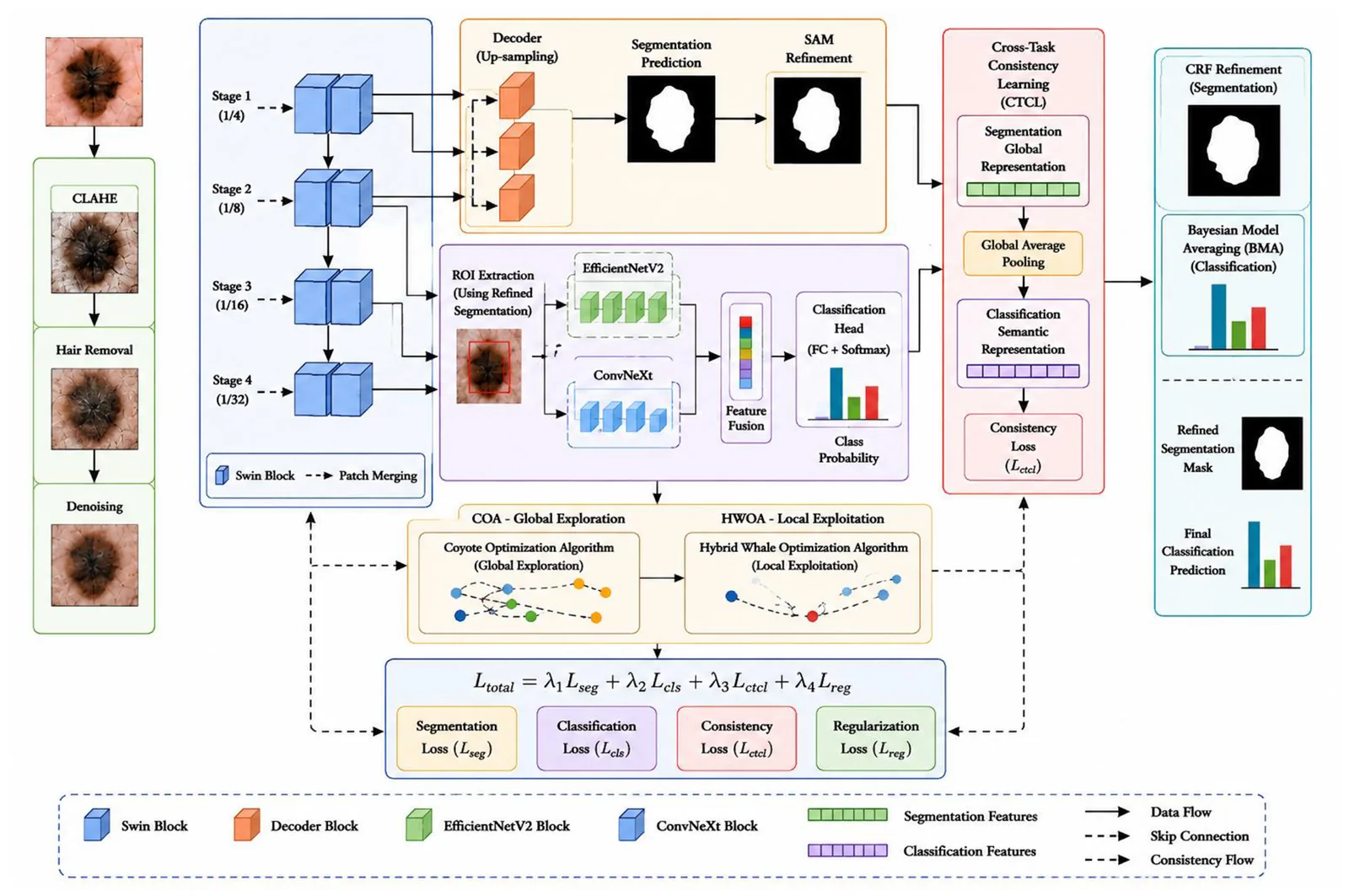
Overall architecture of the proposed SkinNet-X framework.

The pipeline consists of adaptive image enhancement, shared hierarchical feature extraction, dual task learning through lesion segmentation and disease classification branches, Cross Task Consistency Learning (CTCL), training time dual stage feature optimization, joint optimization, and post processing for final skin lesion segmentation and diagnostic classification.

### 3.2 Adaptive image enhancement

Automated skin lesion analysis relies heavily on the quality of dermoscopic pictures. A variety of acquisition abnormalities, including as uneven lighting, poor contrast, hair occlusion, sensor noise, air bubbles, ruler lines, and color discrepancies, regularly affect dermoscopic pictures in real world clinical situations. In addition to impairing lesion border visibility, these artifacts introduce unrelated background information that has a detrimental impact on segmentation and classification performance. As a result, using deep neural networks directly on unprocessed dermoscopic pictures frequently results in unstable illness prediction and incorrect lesion location.

An Adaptive Image Enhancement (AIE) pipeline is used prior to feature extraction in order to mitigate these difficulties. The implied pipeline successively enhances picture quality through three complementary stages local contrast enhancement, artifact suppression, and image denoising instead of depending on a single enhancement approach. The ensuing multitask learning framework may acquire more discriminative spatial and semantic representations while lessening the impact of image artifacts thanks to this sequential improvement. The complete Adaptive Image Enhancement pipeline is illustrated in Figure 2.

**Figure 2:**
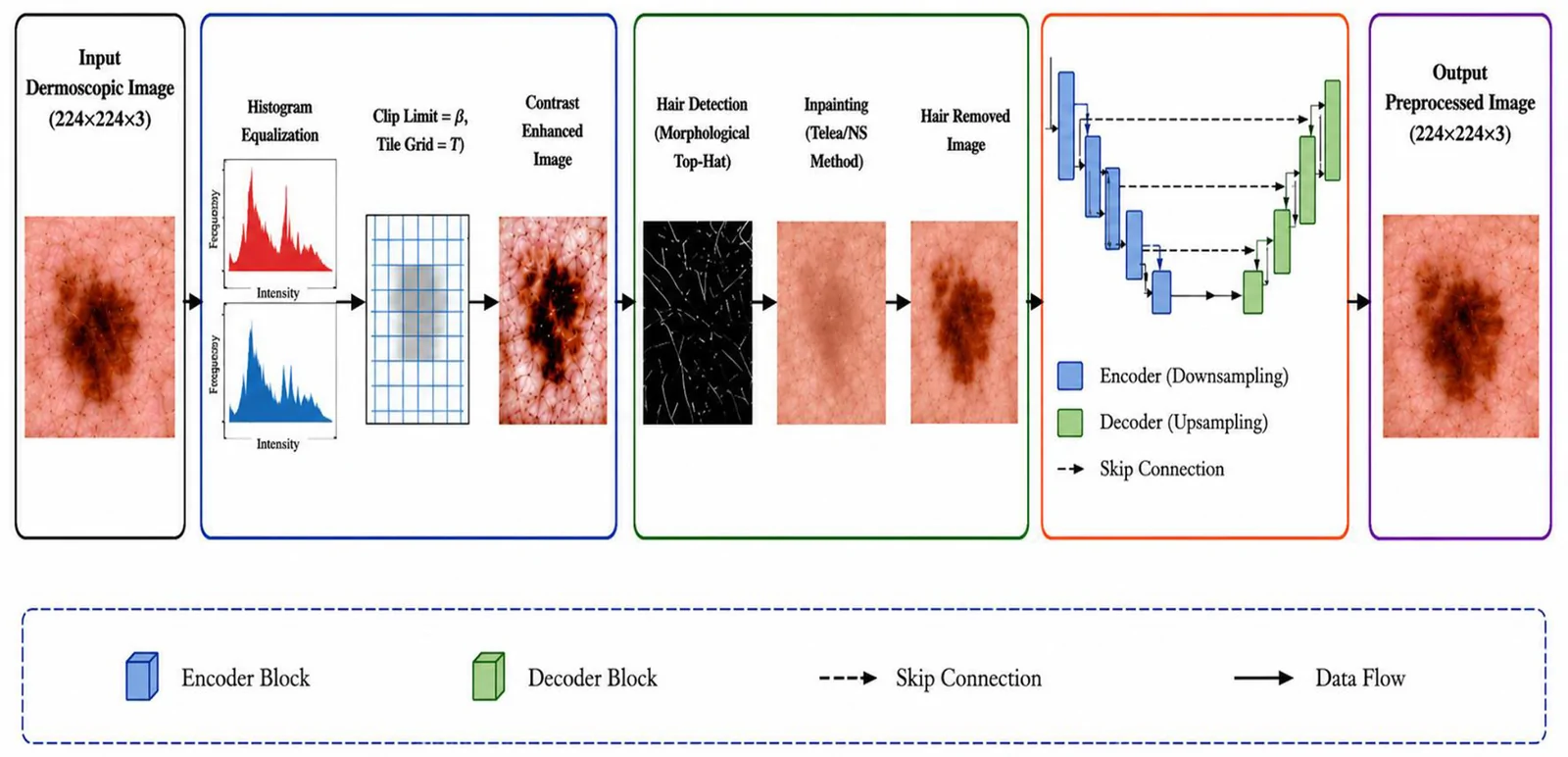
Adaptive image enhancement pipeline (CLAHE → Hair Removal → UNet Denoising).

#### 3.2.1 Local contrast enhancement

By increasing local image contrast, the first step of the proposed preprocessing pipeline seeks to increase lesion visibility. Under non-uniform illumination circumstances, it might be difficult to accurately delineate the boundaries of skin lesions because they often show slight intensity changes from surrounding healthy skin. To improve local intensity changes while avoiding undue amplification of picture noise, Contrast Limited Adaptive Histogram Equalization (CLAHE) is used. In contrast to traditional histogram equalization, which globally redistributes pixel intensities, CLAHE uses contrast clipping to minimize excessive contrast amplification while performing adaptive enhancement within local picture areas. As a result, fine lesion structures become easier to identify without creating unwanted picture aberrations.

Mathematically, the enhanced image can be expressed as


$$\begin{array}{l} I_{enh}=\;CLAHE\left(I_{input}\right) \end{array}$$ (1)

Where, *I*_*input*_ represents the original dermoscopic image and *I*_*enh*_ denotes the locally enhanced image after adaptive histogram equalization.

By highlighting clinically significant features, this improvement increases lesion border visualization and makes future feature extraction easier.

#### 3.2.2 Hair artifact removal

By highlighting clinically significant features, this improvement increases lesion border visualization and makes future feature extraction easier. Because dark hair structures often overlap lesion borders, hair occlusion continues to be a significant difficulty in dermoscopic image processing, leading to inadequate segmentation and deceptive feature extraction. Morphological filtering is first used to detect elongated hair structures in order to minimize these artifacts. Then, using information from nearby pixels, picture inpainting is used to recreate the underlying skin texture. By masking hair sections, the proposed technique reconstructs the affected image regions while keeping lesion shape, hence eliminating structural discontinuities that might adversely affect the segmentation network.

The hair removal process can be represented as


$$\begin{array}{l} I_{hair}=\;Inpaint\left(Morphology\;\left(I_{enh}\right)\right) \end{array}$$ (2)

Where *Morphology*(.) detects hair artifacts and *Inpaint*(.) reconstructs the corresponding image regions. This procedure effectively suppresses hair artifacts while maintaining lesion texture continuity.

#### 3.2.3 Image denoising

Even while contrast enhancement and hair removal significantly improve image quality, differences in light and sensor limits may cause residual acquisition noise to persist. In order to further reduce high frequency noise while maintaining diagnostically significant lesion features, a lightweight UNet based denoising auto-encoder is utilized. Pixel wise reconstruction learning is used to train the denoising network to rebuild clean dermoscopic pictures from noisy inputs. The network improves lesion border continuity without oversmoothing clinically important patterns by maintaining fine structural information during picture restoration.

The denoised image is obtained as


$$\begin{array}{l} {I_{clean}=D}_{\theta }\left(I_{hair}\right) \end{array}$$ (3)

where *D*_θ_(.) denotes the trained denoising auto encoder parameterized by θ.

The proposed denoising technique reduces undesired background changes while better preserving lesion edges as compared to traditional smoothing filters. For later multitask learning, the Adaptive Image Enhancement pipeline offers a number of benefits. First, under different lighting conditions, lesion visibility is improved by local contrast augmentation. Second, hair removal enhances lesion border continuity and lessens interference brought on by occlusion artifacts. Third, picture denoising preserves diagnostically significant structures while suppressing residual acquisition noise. As a result, the improved photos offer a more dependable input for the segmentation and classification branches, which improves optimization stability during multitask learning and allows the proposed SkinNet-X framework to learn more discriminative feature representations.

### 3.3 Shared hierarchical feature representation

When it comes to developing hierarchical representations for medical picture analysis, deep neural networks have shown to be remarkably capable. However, feature extraction is particularly difficult in skin lesion pictures due to significant fluctuations in lesion size, uneven borders, color distribution, textural complexity, and lighting conditions. Fixed receptive fields are often used by conventional convolutional neural networks, which limits their capacity to simultaneously record global contextual linkages and local lesion structures. As a result, feature representations acquired from dermoscopic images might not be enough for precise disease categorization and lesion location. Our SkinNet-X architecture uses a shared hierarchical feature representation as the foundation for both lesion segmentation and illness classification in order to get around these restrictions.

The framework distributes features to both task specific branches after extracting multi scale semantic representations from a common encoder, as opposed to individually learning features for each task. This collaborative learning approach minimizes unnecessary feature extraction while fostering effective knowledge transfer. The proposed architecture incorporates the shared representation with the ensuing Cross Task Consistency Learning (CTCL) module, in contrast to traditional multitask learning frameworks that only share encoder parameters. This enables both downstream tasks to continuously exchange complementary semantic information during optimization. Consequently, the recovered representations simultaneously maintain lesion boundary information essential for segmentation and high level semantic properties required for disease categorization. Because it can describe both local picture structures and long range contextual relationships, a hierarchical vision transformer encoder is used to build this common representation. By dividing the input picture into non-overlapping patches and carrying out hierarchical self attention learning across several stages, the encoder gradually creates multi resolution feature maps. Compared with standard convolutional feature extractors, hierarchical transformers give enhanced contextual modeling while preserving computational efficiency through localized attention computing. The shared hierarchical Swin Transformer backbone and the task-specific segmentation and classification branches are illustrated in Figure 3.

**Figure 3:**
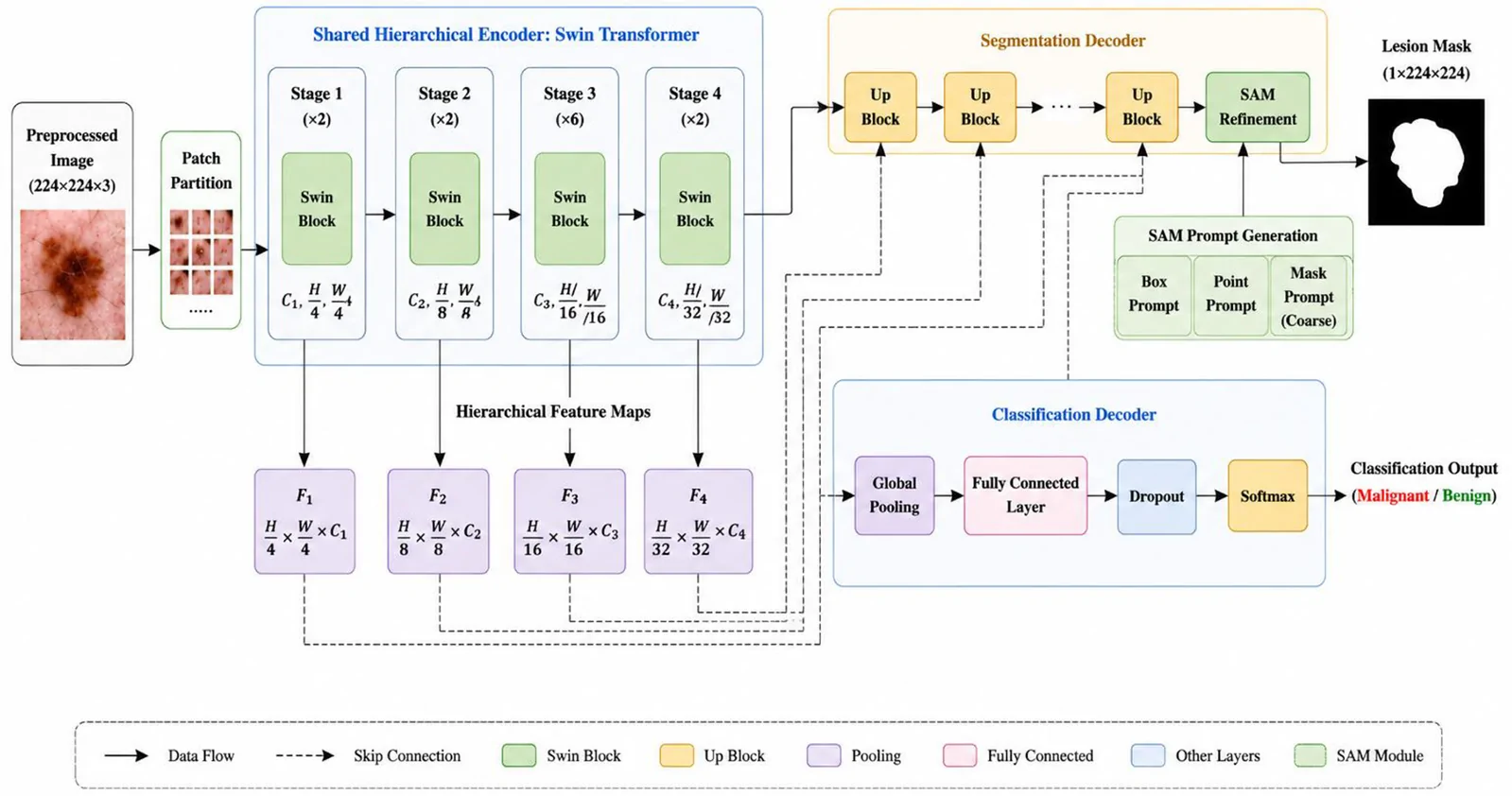
Shared hierarchical Swin transformer backbone with segmentation and classification branches.

The feature extraction process can be represented as


$$\begin{array}{l} F=\Phi \left(I_{clean};\theta_{e}\right) \end{array}$$ (4)

Where,

- *I*_*clean*_ denotes the enhanced dermoscopic image obtained after Adaptive Image Enhancement,
- θ_*e*_ denotes the learnable encoder parameters,
- Φ(·) represents the shared hierarchical encoder,
- F represents the extracted multi scale feature representation.

The extracted feature tensor is subsequently distributed to two parallel learning branches,


$$\begin{array}{l} F_{seg}=\;G_{s}\left(F\right) \end{array}$$ (5)

$$\begin{array}{l} F_{cls}=\;G_{c}\left(F\right) \end{array}$$ (6)

Where, *G*_*s*_ (·) denotes the lesion segmentation branch, *G*_*c*_ (·) denotes the disease classification branch.

Although both branches originate from the same feature hierarchy, they optimize fundamentally different learning objectives. While the classification branch concentrates on image level semantic discrimination, the segmentation branch prioritizes dense pixel level localization. As a result, throughout optimization, the learnt feature distributions progressively diverge in the absence of explicit contact, decreasing cooperation between the two tasks.

In contrast to traditional multitask learning techniques that only use implicit parameter sharing, the approach uses Cross Task Consistency Learning to offer an explicit semantic interaction mechanism. As a result, during network optimization, the shared hierarchical representation serves as both a common feature extractor and an efficient communication bridge that permits ongoing information flow between lesion location and illness detection.

The following succinctly describes the benefits of using a shared hierarchical representation:

- Multi scale contextual information is simultaneously preserved across different network depths.
- Local lesion boundaries and global semantic characteristics are jointly represented.
- Redundant feature extraction between segmentation and classification is eliminated.
- Knowledge transfer between both tasks is naturally facilitated through shared feature learning.
- The shared representation establishes the foundation for the proposed Cross Task Consistency Learning mechanism introduced in the subsequent section.

### 3.4 Dual task learning framework

Our SkinNet-X uses multi-task learning architecture to concurrently segment lesions and classify diseases. Both tasks are concurrently trained using a common hierarchical representation while maintaining task specific learning objectives through dedicated prediction branches, in contrast to traditional methods that independently maximize localization and recognition. By allowing segmentation and classification to share complementing semantic information during training, this collaborative learning approach enhances feature usage and minimizes duplicate representation learning. Despite coming from the same feature encoder, the two activities optimize distinct goals. The segmentation branch stresses on pixel wise lesion localization, whereas the classification branch focuses on discriminative semantic recognition of lesion types. Consequently, separate optimization may ultimately lead to inconsistent feature representations despite having similar encoder settings. In order to overcome this constraint, the two branches are combined into a single optimization framework and then linked via the proposed Cross Task Consistency Learning technique.

#### 3.4.1 Lesion segmentation branch

The precise delineation of lesion borders from dermoscopic images is the responsibility of the lesion segmentation branch. Accurate pixel level prediction needs both local structural knowledge and global contextual awareness since skin lesions often display uneven morphology, fuzzy borders, poor contrast, and large appearance fluctuations. The proposed approach uses a hierarchical transformer based decoder that gradually reconstructs high resolution segmentation masks from the common multi scale feature representation that the encoder generated in order to achieve robust lesion localization. Accurate delineation of complicated lesion patterns is made possible by multi level skip connections, which simultaneously include high level semantic context and maintain precise spatial features. After decoder prediction, a lightweight refinement stage based on the Segment Anything Model (SAM) is added to further improve lesion borders. Instead of carrying out full segmentation on its own, SAM functions as a boundary refinement module that enhances edge continuity and reduces coarse segmentation deviations, especially in the surrounding area of low contrast lesion zones.

Let *F*_*seg*_ denote the segmentation feature representation obtained from the shared encoder. The segmentation prediction is computed as


$$\begin{array}{l} M_{Pred}\;=D\left(F_{seg}\;\right) \end{array}$$ (7)

Where, D(·) represents the segmentation decoder, *M*_*pred*_ denotes the predicted lesion mask. Following decoder prediction, the boundary refinement stage produces


$$\begin{array}{l} M_{ref}\;=SAM\left(M_{pred}\;\right) \end{array}$$ (8)

Where, *M*_*ref*_ represents the refined segmentation mask. The segmentation branch is optimized using pixel wise Binary Cross Entropy (BCE) loss


$$\begin{array}{l} M_{ref}=\frac{1}{N}\sum_{n=1}^{\infty }\left[y_{i}\log(\hat{yi})+(1-y_{i})\log(1-\hat{yi})\right] \end{array}$$ (9)

Where, *y*_*i*_ denotes the ground truth pixel, $\hat{y_{i}}$ denotes the predicted probability. The refined segmentation representation is subsequently forwarded to the proposed CTCL module, allowing lesion localization to participate directly in semantic consistency learning rather than functioning as an isolated prediction task.

#### 3.4.2 Disease classification branch

The shared hierarchical encoder's semantic representation is used by the disease classification branch to anticipate the segmented lesion's diagnostic category. By using the spatial information supplied by the segmentation branch, the proposed method stresses lesion focused feature learning rather than directly classifying the full dermoscopic picture. As a result, background interference is significantly decreased, allowing the classifier to focus on lesion shapes that are clinically significant. Complementary deep feature extractors that work together to jointly learn discriminative semantic properties from lesion focused areas make up the classification branch. Before being transmitted to the last classification layer, these representations are then combined via global feature pooling.

The classification prediction is expressed as


$$\begin{array}{l} P_{cls}=C\left(F_{cls}\right) \end{array}$$ (10)

$$\begin{array}{l} F_{pool}=GAP\left(F_{seg}\right) \end{array}$$ (11)

Denotes classification features, C(·) denotes the classification network, *P*_*cls*_ represents the predicted probability distribution over disease categories. The classification objective is optimized using PolyLoss enhanced cross entropy learning


$$\begin{array}{l} L_{cls}\;=-\overset{K}{\underset{c=1}{\sum }}y_{c}\log\left(\hat{yc}\right)+\epsilon \left(1-\hat{yc}\right) \end{array}$$ (12)

Where, *K* denotes the number of lesion categories, *y*_*c*_ denotes the ground truth class, $\hat{y_{c}}$ denotes the predicted probability, ϵ represents the polynomial correction coefficient.

PolyLoss offers more optimization flexibility than traditional cross entropy optimization, which enhances convergence stability during multitask learning while preserving computing efficiency. Dissimilarly conventional standalone classifiers, the proposed classification branch continuously interacts with lesion localization through the subsequent Cross Task Consistency Learning module, enabling disease prediction to remain semantically consistent with lesion boundary information throughout network optimization.

### 3.5 Cross task consistency learning (CTCL)

While multitask learning has emerged as a successful paradigm for **concurrently carrying out** lesion segmentation and disease classification, the majority of current frameworks establish interaction between multiple tasks through shared encoder parameters, feature concatenation and attention based feature fusion. These strategies implicitly assume that both tasks naturally learn compatible semantic representations during optimization, but this assumption rarely holds in practice because lesion segmentation and disease classification optimize fundamentally different objectives: classification seeks to discriminate image level semantic categories, while segmentation focuses on dense pixel wise localization. As a result, the learned feature distributions gradually diverge during training, resulting in cross task semantic inconsistency that limits collaborative learning.

The proposed Cross Task Consistency Learning (CTCL) technique is presented to explicitly regularize the semantic interaction between illness categorization and lesion segmentation in order to overcome this constraint. Throughout end-to-end optimization, CTCL constantly pushes both prediction branches to acquire mutually compatible semantic knowledge rather than only exchanging intermediate feature representations. As a result, while disease semantics concurrently offer supervisory direction for lesion localization, lesion localization directly contributes to illness detection. In contrast to traditional feature-sharing techniques that rely primarily on implicit information exchange, CTCL introduces explicit cross-task semantic regularization between the projected segmentation and classification representations. This explicit contact minimizes optimization conflicts resulting from varied prediction tasks while enabling both learning targets to grow cooperatively.

#### 3.5.1 Motivation

Following the shared encoder, the segmentation and classification branches are tuned separately in traditional multitask learning. Both branches progressively acquire task specific representations that optimize their respective goal functions as training goes on. Pixel level segmentation and image level classification need distinct semantic qualities, therefore even though both tasks start from the same encoder features, their optimal trajectories gradually diverge. Inconsistent feature representations result from segmentation's emphasis on border localization and classification's emphasis on illness semantics. This semantic discrepancy restricts the potential benefits of multitask learning and diminishes the efficacy of feature sharing.

In order to encourage both branches to grow toward mutually compatible semantic representations, the proposed CTCL adds an extra semantic consistency constraint that continually decreases the difference between the two task representations throughout optimization.

#### 3.5.2 Cross task representation alignment

Let $F_{seg}\;{\mathbb{R}}^{H*W*}$C denote the feature representation produced by the lesion segmentation branch.

Similarly, $F_{cls}\;{\mathbb{R}}^{C}$ represents the semantic representation learned by the disease classification branch. Since both representations possess different spatial dimensions, the segmentation feature map is transformed into a compact semantic descriptor using Global Average Pooling


$$\begin{array}{l} F_{pool}=GAP\left(F_{seg}\right) \end{array}$$ (13)

Where, $(F_{pool})\;{\mathbb{R}}^{C}$ this transformation converts pixel level spatial representations into a global semantic representation having the same dimensionality as the classification feature vector. Subsequently, both representations are projected into a common semantic embedding space,


$$\begin{array}{l} Z_{s}=\phi_{S}(F_{pool}) \end{array}$$ (14)

$$\begin{array}{l} Z_{c}=\phi_{c}(F_{cls}) \end{array}$$ (15)

Where, ϕ_*S*_ (.) and ϕ_*c*_ (.) denote learnable projection functions implemented using lightweight fully connected layers.

#### 3.5.3 Cross task consistency loss

After obtaining both semantic embeddings, semantic disagreement between segmentation and classification is minimized through the proposed consistency objective


$$\begin{array}{l} F_{opt}=\;\frac{1}{N}\overset{N}{\underset{i=1}{\sum }}|Z\begin{array}{c} \left(i\right)\\ C \end{array}-\;Z\begin{array}{c} \left(i\right)\\ C \end{array}|\begin{array}{c} 2\\ 2 \end{array} \end{array}$$ (16)

Where, *N* denotes batch size, *Z*_*s*_ represents segmentation embedding, *Z*_*C*_ represents classification embedding. While maintaining their distinct learning goals, the proposed framework promotes lesion localization and illness detection to provide semantically consistent representations. In contrast to direct feature concatenation, CTCL does not require both jobs to be the same. Rather, it avoids needless semantic dispute while preserving discriminative information distinct to each branch. It should be emphasized that CTCL does not require the original segmentation and classification feature representations to become identical. Both branches retain their task-specific feature spaces and continue to be optimized using their respective segmentation and classification objectives. The consistency constraint is applied only after independent projection of the two representations into a shared latent semantic space. Consequently, CTCL acts as a soft representation-level regularizer that encourages compatibility between lesion-related semantic information while preserving task-specific spatial and diagnostic characteristics. Therefore, a reduction in CTCL loss is interpreted as reduced disagreement between the projected task representations rather than complete semantic equivalence between the heterogeneous tasks.

#### 3.5.4 Difference from existing consistency learning

Several consistency learning strategies have been reported in recent literature, including Kullback-Leibler (KL) divergence, contrastive representation learning, mutual information maximization, and teacher student consistency learning. Although these approaches have demonstrated promising performance in semi-supervised learning and domain adaptation, they primarily enforce consistency within a single prediction objective. In contrast, the proposed CTCL is specifically designed for cross task semantic consistency between heterogeneous learning objectives. Dissimilar KL-divergence regularization, which aligns probability distributions, CTCL aligns semantic feature representations generated from fundamentally different prediction tasks. Similarly, contrastive learning maximizes representation similarity between positive sample pairs while separating negative pairs. However, the objective of CTCL is not representation discrimination but semantic cooperation between lesion localization and disease recognition.

Additionally, by continuously regularizing the interaction between segmentation and classification throughout network optimization, CTCL directly addresses the issue of mutual information maximization, which aims to preserve shared information between representations without explicitly minimizing semantic disagreement arising from heterogeneous task objectives. Consequently, CTCL should be considered as a multi-task semantic consistency mechanism rather than a traditional consistency learning strategy. A conceptual comparison of CTCL with existing multitask and consistency learning strategies is presented in Table 2.

**Table 2:** Conceptual comparison of CTCL with existing multitask and consistency learning strategies.

Method	Main principle	Interaction type	Limitation	Difference of proposed CTCL
Shared encoder multitask learning	Common backbone is shared by tasks	Implicit feature sharing	Does not explicitly reduce semantic mismatch	CTCL directly aligns segmentation and classification representations
Attention based fusion	Attention weights combine task features	Feature level fusion	Improves feature aggregation but not semantic consistency	CTCL regularizes cross task semantic agreement
Auxiliary task learning	One task supports another through extra supervision	Indirect task supervision	Auxiliary task may not enforce agreement with main task	CTCL jointly constrains both tasks during optimization
KL consistency	Aligns probability distributions	Distribution level alignment	Usually assumes similar output spaces	CTCL aligns heterogeneous segmentation/classification embeddings
Contrastive learning	Pulls positives closer and pushes negatives apart	Representation discrimination	Requires positive/negative pair design	CTCL focuses on cooperative task agreement
Mutual information optimization	Maximizes shared information	Information preservation	Does not directly minimize task disagreement	CTCL minimizes cross task semantic discrepancy

Compared to traditional multitask learning frameworks, the proposed Cross Task Consistency Learning offers a number of significant advantages.

- The explicit semantic relationship between illness identification and lesion location.
- Less conflict between various learning targets during optimization.
- Semantic alignment improves feature discriminability.
- Improved optimization stability during the whole multitask learning process.
- Improved cooperation between the classification and segmentation branches.
- Since CTCL is only active during training, there is no additional computing cost during inference.

#### 3.5.5 Algorithm of CTCL

Input: Dermoscopic images with task-specific segmentation masks and diagnostic class labels.

Output: Optimized SkinNet-X model.

1. Initialize SkinNet-X parameters.
2. Preprocess dermoscopic images.
3. Extract hierarchical features using Swin Transformer.
4. Generate segmentation prediction.
5. Refine lesion boundaries using SAM.
6. Generate lesion-guided classification features.
7. Generate disease-class prediction.
8. Establish cross-task representations using CTCL.
9. Compute segmentation, classification and CTCL losses.
10. Apply PolyLoss to the classification objective.
11. Perform COA-based feature exploration.
12. Perform HWOA-based feature refinement.
13. Update model parameters using AdamW.
14. Repeat Steps 2–12 until the specified epochs are completed.
15. Apply CRF post-processing.
16. Apply BMA for final classification.
17. Return the optimized SkinNet-X model.

### 3.6 Training time dual stage feature optimization

Not every extracted feature contributes equally to lesion segmentation and disease classification, despite the fact that deep neural networks automatically learn hierarchical feature representations. Redundant, correlated, and less informative features may reduce optimization efficiency, increase computational complexity, and negatively influence generalization performance. This problem becomes more pronounced in multitask learning, where segmentation and classification simultaneously compete for shared feature representations during network optimization. To alleviate this limitation, the proposed SkinNet-X incorporates a training time dual stage feature optimization strategy that combines global exploration and local exploitation to improve feature quality before final model convergence. Dissimilarly conventional gradient based optimization, which updates network parameters solely according to the loss gradient, the proposed optimization framework introduces complementary search mechanisms that improve feature selection and optimization stability during network training.

The proposed optimization strategy consists of two sequential stages.

#### 3.6.1 Motivation for guided multi-task optimization

In conventional multitask learning, segmentation and classification are optimized simultaneously through shared representations and task-specific objectives. However, the two tasks may follow different optimization directions because segmentation emphasizes spatial lesion localization, whereas classification emphasizes disease-discriminative semantic information. Therefore, simply sharing features does not necessarily ensure that the learned representation is optimal for both tasks. Recent theoretical work on Mentored Learning has shown that guided learning based on an approximately optimal teaching hypothesis can improve the convergence and generalization behavior of a learner (Cao et al., 2024). Inspired by this general principle of guided optimization, SkinNet-X incorporates a training-time dual-stage feature optimization strategy to guide the shared representation toward feature configurations that are more suitable for the coupled segmentation and classification objectives. In the proposed framework, CTCL provides the cross-task semantic consistency signal, while COA performs global feature exploration and HWOA subsequently performs local feature refinement. Thus, the optimization process is guided by the joint multitask objective rather than relying solely on unconstrained gradient-based parameter updates. Unlike Mentored Learning, which explicitly considers a teacher–learner setting, SkinNet-X operates in a supervised multitask medical-image setting and does not introduce an external teacher model. The Mentored Learning concept is therefore used as theoretical motivation for guided optimization rather than as a direct implementation of the proposed framework.

#### 3.6.2 Global feature exploration

In the first stage, the Coyote Optimization Algorithm (COA) performs global exploration over the learned feature space to identify informative feature subsets that contribute most effectively to both lesion localization and disease recognition. The population based search strategy enables the optimization process to escape local optima while maintaining feature diversity during early network training. Let *F* = {*f*1, *f*2, …., *fm*} denote the extracted feature representation. The optimized feature subset can be expressed as


$$\begin{array}{l} F_{opt}=COA\;\left(F\right) \end{array}$$ (17)

Where, *F*_*opt*_ represents the globally optimized feature representation obtained through population based exploration.

#### 3.6.3 Local feature refinement

Compact and discriminative feature representations require more refinement, even though global exploration increases feature diversity. Consequently, the Hybrid Whale Optimization Algorithm (HWOA) is used to further refine the optimized feature subset produced by COA. By continuously improving feature selection in accordance with the optimization objective, HWOA executes adaptive local exploitation around promising feature candidates. This improvement decreases redundancy between correlated feature representations while increasing feature compactness.

The refined feature representation is computed as


$$\begin{array}{l} F_{opt}=HWOA\left(F_{COA}\right) \end{array}$$ (18)

The final optimized feature representation utilized during network training is indicated by *F*_*opt*_. The proposed optimization strategy constantly looks for better feature representations during training, allowing the multitask network to converge toward more discriminative semantic representations, in contrast to traditional feature selection techniques that permanently remove features.

The proposed optimization strategy provides several advantages:

- Improves feature discriminability.
- Reduces redundant feature representations.
- Enhances convergence stability.
- Prevents premature convergence.
- Improves multitask learning efficiency.
- Does not increase computational cost during inference because optimization is performed only during training.

The dual-stage feature search is complementary to, rather than a replacement for, gradient-based network training. AdamW and backpropagation update the learnable network parameters according to the joint loss, whereas COA-HWOA operates on the intermediate feature optimization process during training. The optimized feature configuration is subsequently used by the network while parameter learning continues through gradient descent. Thus, population-based feature search and gradient-based parameter optimization perform distinct but coordinated roles.

### 3.7 Joint optimization strategy

Within a single end-to-end learning framework, the SkinNet-X concurrently optimizes lesion segmentation, illness classification, semantic consistency, and network regularization. A composite loss function that promotes cooperative learning across the two prediction tasks is used to jointly maximize all learning components rather than maximizing each goal separately.

The overall optimization objective is formulated as


$$\begin{array}{l} L_{Total}\;=\;\lambda_{seg}L_{seg}\;{+\lambda }_{cls}L_{cls}+\lambda_{CTCL}L_{CTCL}+\lambda_{reg}L_{reg} \end{array}$$ (19)

Where, *L*_*leg*_ represents the segmentation loss, *L*_*cls*_ denotes the classification loss, *L*_*CTCL*_ corresponds to the proposed Cross Task Consistency Learning loss, *L*_*reg*_ denotes regularization loss, are balancing coefficients controlling the contribution of each learning objective.

λ_*leg*_, λ_*cls*_, *L*_*CTCL*_, λ_*reg*_ CTCL reduces semantic disagreement across both prediction branches, regularization suppresses overfitting, segmentation supervision increases lesion localization accuracy, and classification supervision improves disease discrimination during optimization. As a result, rather than competing individually, all learning objectives develop together throughout network training. The AdamW optimizer with adaptive learning rate scheduling is used to carry out the optimization procedure.

Network parameters are updated through backpropagation until convergence according to where


$$\begin{array}{l} \theta^{t+1}=\theta^{t}-\eta \nabla L_{Total} \end{array}$$ (20)

θ denotes network parameters, η represents learning rate. The proposed unified optimization strategy continuously coordinates segmentation, classification, and semantic consistency learning within a single optimization process, improving convergence stability and multitask collaboration in contrast to traditional multitask learning frameworks that independently optimize multiple objectives.

### 3.8 Post processing and final prediction

Lightweight post processing is used after network inference to strengthen the robustness of illness prediction and improve the visual quality of lesion segmentation.

#### 3.8.1 Segmentation refinement

Although the segmentation branch provides accurate lesion masks, slight boundary discontinuities may still exist around low contrast lesion locations. Consequently, a boundary refinement approach called Conditional Random Fields (CRFs) is used. CRF preserves fine lesion borders while enhancing pixel label consistency through the use of neighborhood spatial interactions.

The refined segmentation mask is represented as


$$\begin{array}{l} M_{final}=CRF\;\left(M_{ref}\right) \end{array}$$ (21)

Where, *M*_*ref*_ denotes the refined prediction obtained after decoder inference. The incorporation of CRF improves lesion boundary continuity while suppressing isolated segmentation artifacts.

#### 3.8.2 Classification refinement

Bayesian Model Averaging (BMA) is used to increase prediction robustness for illness prediction. BMA reduces the uncertainty associated with individual predictions by combining probabilistic outcomes from several learned hypotheses rather than depending solely on one.

The final prediction probability is computed as


$$\begin{array}{l} P_{final}=\underset{i=1}{\sum }w_{i\;}P_{i} \end{array}$$ (22)

Where, *P*_*i*_ represents the probability estimated by the *i*^*th*^ predictive model, and $w_{i}$ denotes its corresponding Bayesian weight. This probabilistic fusion lessens susceptibility to model uncertainty and increases prediction stability. Refined lesion segmentation, disease classification probability, and lesion diagnostic category make up SkinNet-X's final output. Because optimization algorithms are only used during network training and the deployed inference model only includes the trained segmentation and classification networks together with lightweight refinement modules, the inference pipeline as a whole remains computationally efficient.

## 4 Experimental setup

The publicly accessible ISIC 2018, ISIC 2019, and HAM10000 datasets were used for the experimental evaluation. ISIC 2018 was used for supervised lesion segmentation, whereas ISIC 2019 and HAM10000 were used for multiclass disease classification. To guarantee fair comparison across trials, the datasets were separated into subsets for training, validation, and testing using fixed splits.

### 4.1 Datasets

The experiments were conducted on ISIC 2018, ISIC 2019, and HAM10000 datasets. The characteristics of the datasets are summarized in Table 3.

**Table 3:** Overview of publicly available dermoscopic datasets used for experimental evaluation.

Dataset	Images	Classes	Resolution	Task
ISIC 2018	2,594	Binary	512 × 512	Segmentation
ISIC 2019	25,331	8	Variable	Classification
HAM10000	10,015	7	600 × 450	Classification

#### 4.1.1 Task-specific supervision strategy

The benchmark datasets provide complementary, task-specific forms of supervision rather than artificially paired annotations across datasets. ISIC 2018 provides pixel-level lesion-mask supervision for the segmentation objective, whereas ISIC 2019 and HAM10000 provide diagnostic class-label supervision for the classification objective. No segmentation masks are assumed for ISIC 2019 or HAM10000, and no segmentation ground-truth evaluation is performed on these classification datasets. The segmentation and classification branches retain their respective task-specific supervision, while CTCL operates at the learned representation level to encourage compatibility between lesion-related and diagnostic semantic representations. Thus, joint optimization refers to coordinated optimization of the two task objectives within the unified SkinNet-X framework rather than synthetic cross-dataset annotation pairing.

For each dataset, a fixed data splitting strategy was used to provide a reliable and reproducible evaluation of the proposed framework. The dataset split configuration adopted for training and testing is presented in Table 4.

**Table 4:** Training, validation, and testing split ratios adopted for each dataset.

Dataset	Train	Validation	Test
ISIC 2018	70%	10%	20%
ISIC 2019	70%	10%	20%
HAM10000	75%	10%	15%

### 4.2 Data partitioning

See Table 4 for Data partitioning.

### 4.3 Implementation details

The proposed SkinNet-X framework was built using Python 3.12 and PyTorch 2.x on the Google Colab platform with CUDA enabled GPU acceleration. The network was trained for 150 epochs using the AdamW optimizer with a batch size of 16 and an initial learning rate of 1 × 10?^6^. To increase optimization stability during training, a cosine annealing learning-rate scheduler was used. All dermoscopic pictures were scaled to 224 × 224 pixels before training. During training, online data augmentation methods such as random rotation, brightness modification, horizontal flipping, vertical flipping, and normalization were used to enhance model generalization and decrease overfitting. The random seed was set at 42 to guarantee experimental repeatability, and each experiment was conducted three times independently using the same training parameters.

The mean ± standard deviation from these separate runs matches the reported performance. The transformer based backbone was implemented using the TIMM library, while the computational complexity of the proposed method, including the total number of trainable parameters and floating point operations (FLOPs), was estimated using THOP. Additionally, single image forward propagation was used to determine the average inference time and frames per second (FPS) in order to assess deployment efficiency. The unified objective function outlined in Section 3.7 was used to jointly optimize the segmentation loss, classification loss, Cross Task Consistency Learning (CTCL) loss, and regularization loss during end-to-end multitask learning. Figure 4 displays representative dermoscopic picture samples from the benchmark datasets.

**Figure 4:**
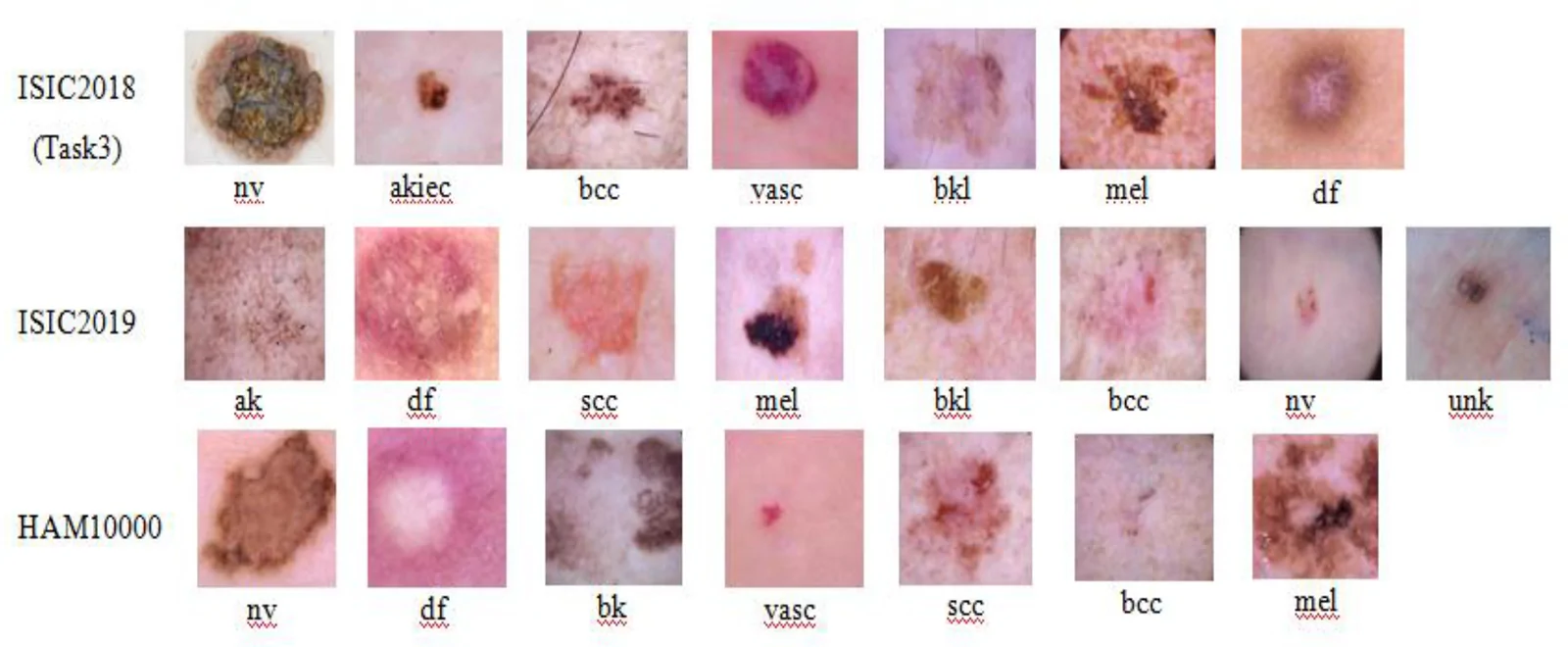
Representative class wise sample images from the datasets.

The implementation source code and trained model weights will be made freely accessible upon publication or upon reasonable request in order to promote reproducibility and future study. Table 5 reports the loss-weight scheduling strategy adopted during the final joint-training stage of SkinNet-X. In contrast, Table 6 represents a separate controlled sensitivity experiment in which λCTCL is held fixed at different values while the remaining training configuration is kept unchanged. The purpose of Table 6 is therefore to study sensitivity to consistency strength rather than to describe the dynamic schedule used for the final reported model.

**Table 5:** Dynamic loss weight scheduling during joint optimization.

Epoch	λSeg	λCls	λCTCL	λReg
10	0.80	0.10	0.05	0.05
25	0.70	0.15	0.10	0.05
50	0.60	0.20	0.15	0.05
75	0.55	0.25	0.15	0.05
100	0.50	0.30	0.15	0.05
125	0.45	0.35	0.15	0.05
150	0.40	0.40	0.15	0.05

**Table 6:** Sensitivity analysis of CTCL weight.

λCTCL	Dice (%)	IoU (%)	Classification accuracy (%)
0.0	93.28	87.95	95.84
0.1	93.76	88.67	96.71
**0.3**	**95.12**	**90.18**	**97.12**
0.5	94.17	89.24	96.84
0.7	93.81	88.75	94.96

Bold values indicate the best performance obtained across the evaluated CTCL weight settings.

### 4.4 Computational complexity analysis

The proposed SkinNet-X framework's computational efficiency was examined in addition to its segmentation and classification capabilities. The THOP profiling package was used to quantify the total number of trainable parameters and floating point operations (FLOPs) in order to assess model complexity. Additionally, utilizing a single input picture, inference speed was calculated by averaging the processing time across many forward passes. These calculations offer an estimate of the computing cost and illustrate the viability of implementing the proposed framework in real time clinical situations. The computational complexity measurements of the proposed SkinNet-X framework are summarized in Table 7.

**Table 7:** Computational Complexity of SkinNet-X.

Metric	Value
Total parameters	14.45 M
Trainable parameters	14.45 M
FLOPs	4.78 G
Training time (150 epochs)	17,850 s
Average inference time	8.90 ms
FPS	112.36

The reported parameter count and FLOPs were obtained using THOP for the core trainable neural-network architecture of SkinNet-X. Similarly, the reported average inference time and FPS correspond to repeated forward propagation through the profiled neural model. These measurements do not include offline/adaptive preprocessing or external post-processing operations such as CRF. Therefore, the reported 4.78 GFLOPs, 8.90 ms inference time, and 112.36 FPS should be interpreted as core-model computational measurements rather than complete preprocessing-to-post-processing pipeline latency. This distinction provides a more transparent assessment of deployment efficiency.

First, the segmentation network was trained separately using binary cross entropy loss and dice loss to learn lesion borders. Features taken from the segmentation backbone were used to train the classification branch after stable segmentation performance was attained. Both branches were simultaneously fine tuned in the last stage. Dynamic loss weighting prevented either task from controlling the optimization process during joint training by adjusting each task's contribution based on relative loss behavior. Table 5 presents the dynamic loss-weight scheduling strategy adopted during the final joint-training stage of SkinNet-X.

### 4.5 Sensitivity analysis of CTCL weight

In addition to determining the best weighting technique for striking a balance between segmentation and classification goals, this experiment explores whether the model's performance is stable under varying degrees of consistency regularization.

The findings show that the optimal balance between segmentation and classification is achieved with modest CTCL regularization. While excessively large weights marginally diminish task specific learning capacity, excessively small weights are unable to take advantage of semantic consistency. To evaluate the effect of the proposed Cross Task Consistency Learning loss, the weighting coefficient λCTCL was adjusted while retaining similar training conditions. To isolate the influence of the CTCL regularizer, λCTCL = 0.0 is treated as the CTCL-OFF configuration, while all remaining architectural and training conditions are retained. The non-zero settings correspond to CTCL-enabled configurations with progressively stronger consistency regularization. The comparison therefore provides a controlled assessment of the effect of introducing CTCL without modifying the underlying multitask architecture. The results show that moderate CTCL regularization improves both segmentation and classification relative to the CTCL-OFF condition, whereas excessively high consistency weighting can restrict task-specific learning.

### 4.6 Training convergence analysis

The proposed SkinNet-X framework's convergence behavior during multitask optimization. Throughout the training process, the overall training loss gradually drops, demonstrating consistent and efficient optimization with no discernible oscillations (Figure 5).

**Figure 5:**
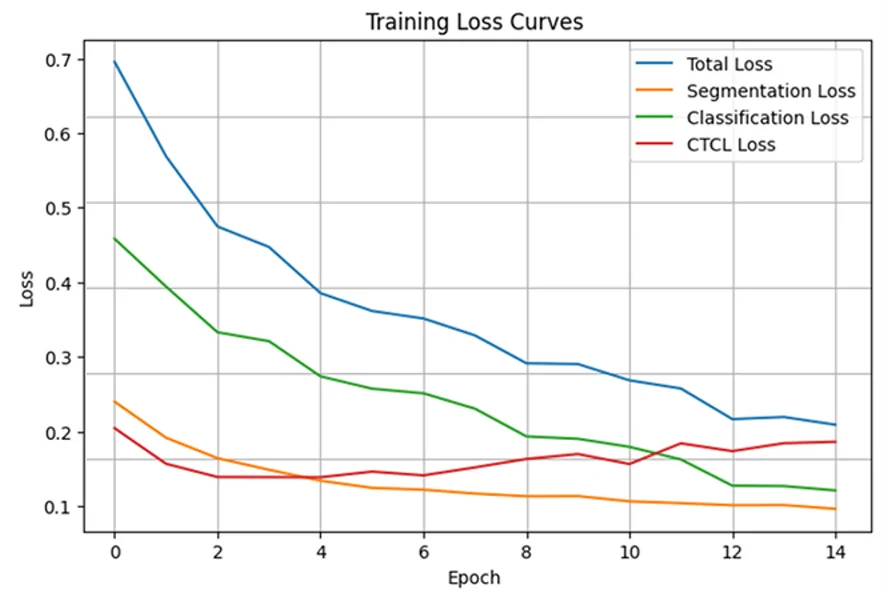
Illustrates the convergence behavior of the proposed SkinNet-X during multitask optimization.

The segmentation loss demonstrates a quick drop during the initial training epochs owing to the effective learning of lesion border representations. In a similar vein, as the network gradually acquires discriminative semantic information for lesion classification, the classification loss steadily declines. The proposed Cross Task Consistency Learning (CTCL) loss stays constant after the early epochs, suggesting good semantic alignment between the segmentation and classification branches. Overall, the convergence curves show that the proposed joint optimization approach successfully accomplishes steady end-to-end training while balancing various learning objectives. The sensitivity and convergence analyses provide empirical evidence regarding the influence and stability of CTCL during joint optimization. However, these performance-based results should not be interpreted as direct measurement of complete latent-space equivalence between the two tasks. A dedicated representation-space analysis using cosine similarity or embedding-distance trajectories would provide additional direct evidence and is considered an important extension for future investigation.

### 4.7 Segmentation performance

The segmentation performance of the proposed SkinNet-X framework was assessed using Dice coefficient, Intersection over Union (IoU), Pixel Accuracy, Precision, Recall, and Area Under the ROC Curve (AUC) on the ISIC 2018 dataset. Table 8 summarizes the quantitative segmentation performance obtained on ISIC 2018. The proposed framework achieved a Dice coefficient of 95.12%, an IoU of 90.18%, Pixel Accuracy of 98.74%, Precision of 97.11%, Recall of 98.64%, and an AUC of 99.86%. These results demonstrate accurate lesion boundary localization and strong agreement between predicted and ground-truth masks.

**Table 8:** Summary of segmentation performance metrics of the proposed model on the ISIC 2018 dataset.

Dataset	Dice	IoU	Pixel accuracy	Precision	Recall	AUC
ISIC2018	95.12	90.18	98.74	97.11	98.64	99.86

The experimental findings show that the proposed SkinNet-X framework produces accurate lesion segmentation on the ISIC 2018 benchmark dataset. While the high Pixel Accuracy, Precision, Recall, and AUC prove dependable lesion localization under various lesion sizes, forms, textures, and lighting circumstances, the high Dice coefficient and IoU show great agreement between the predicted lesion masks and the ground truth annotations. The proposed Cross Task Consistency Learning (CTCL) mechanism, which promotes semantic cooperation between segmentation and classification during joint optimization, SAM based boundary refinement for maintaining fine lesion structures, and the complementary integration of the Swin Transformer encoder for multi scale contextual representation are responsible for the enhanced segmentation performance. These findings show that explicit cross task semantic interaction outperforms traditional implicit feature sharing techniques in terms of lesion border delineation accuracy and robustness. Qualitative segmentation results produced by the proposed SkinNet-X framework are shown in Figure 6.

**Figure 6:**
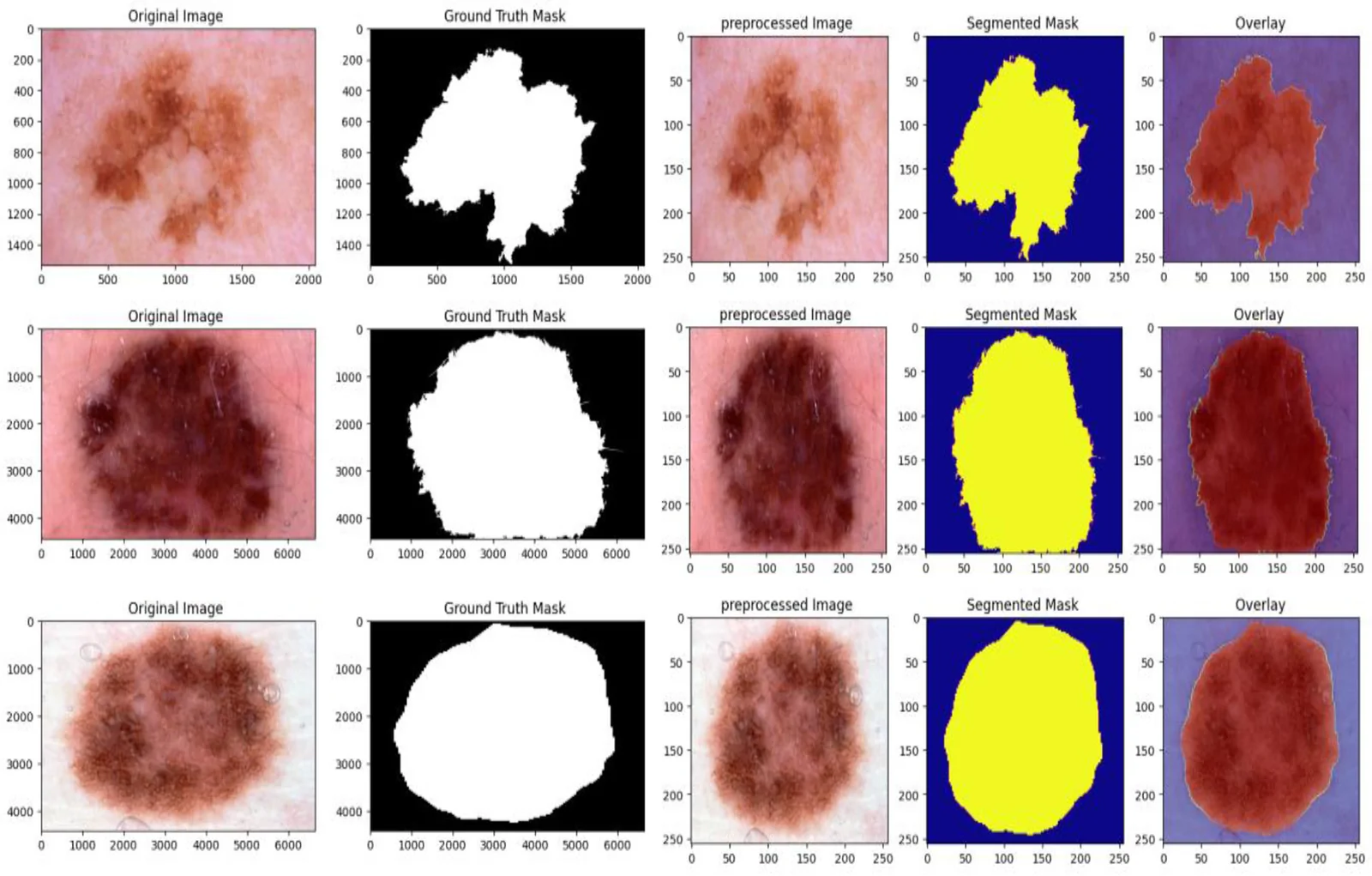
Visual comparison of segmentation results produced by the proposed SkinNet-X model on representative ISIC 2018 test images.

The proposed SkinNet-X framework generates representative qualitative segmentation results. While properly maintaining irregular lesion borders, fine features, and lesion morphology, the predicted lesion masks closely resemble the manually annotated ground truth masks. The overlay visualizations further validate the efficacy of the proposed segmentation framework by showing accurate border localization with few false positive and false negative areas. The segmentation performance metrics observed during training are presented in Figure 7.

**Figure 7:**
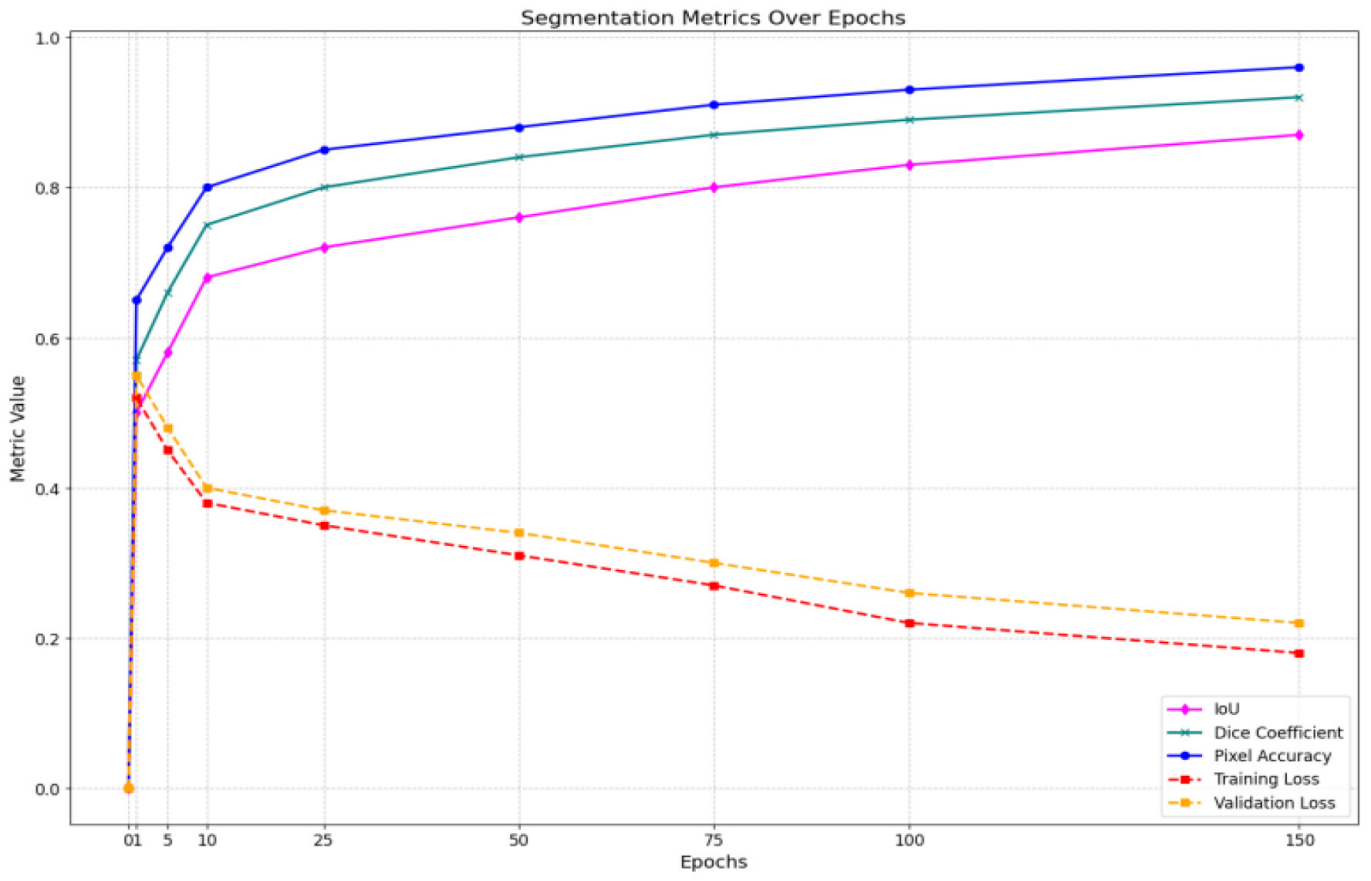
Segmentation performance metrics during training on the ISIC 2018 dataset.

### 4.8 Classification performance

The proposed SkinNet-X framework's classification performance was assessed using Accuracy, Precision, Recall, F1-score, and Area Under the ROC Curve (AUC) on the ISIC 2019 and HAM10000 datasets. Table 9 displays the quantitative findings. The proposed method produced an overall classification accuracy of 98.03% on the ISIC 2019 dataset, along with a precision of 97.71%, recall of 98.54%, F1-score of 98.11%, and AUC of 99.24%. Comparably, the proposed framework demonstrated dependable multiclass lesion detection with an overall classification accuracy of 98.46% on the HAM10000 dataset while retaining good Precision, Recall, and F1-score values. Segmentation-guided feature extraction contributes to improved classification by encouraging the classification branch to focus on lesion-specific regions rather than surrounding healthy skin. However, because the complete SkinNet-X framework contains multiple interacting components, the observed classification improvement should be interpreted as the combined effect of lesion guidance, CTCL regularization, feature optimization, and PolyLoss rather than being attributed exclusively to segmentation guidance. Additionally, the COA-HWOA feature optimization technique boosts discriminative feature learning, PolyLoss stabilizes multitask optimization, and the proposed CTCL mechanism improves semantic consistency between lesion location and illness detection.

**Table 9:** Overall classification performance of the proposed SkinNet-X framework across ISIC 2019 and HAM10000 datasets.

Dataset	Accuracy	Precision	Recall	F1	AUC
ISIC2019	98.03	97.71	98.54	98.11	99.24
HAM10000	98.46	98.21	98.39	98.30	99.51

The classification findings show that the proposed SkinNet-X architecture achieves high aggregate Precision, Recall, F1-score, and AUC values while offering dependable illness detection across several benchmark datasets. Dissimilarly traditional image level classification approaches, The proposed methodology employs lesion guided semantic representations produced from the segmentation branch, allowing the classifier to focus on clinically significant lesion locations while reducing background interference. Additionally, the proposed CTCL process improves optimization stability and more discriminative feature learning by strengthening semantic coherence between lesion location and illness detection. These elements support consistent generalization across various dermoscopic datasets and enhanced classification accuracy when combined with the training time dual stage feature optimization technique and PolyLoss optimization. A quantitative comparison of the classification performance across the ISIC 2019 and HAM10000 datasets is shown in Figure 8. The corresponding confusion matrices for multiclass classification are presented in Figure 9. The training and validation accuracy and loss curves over 150 epochs are shown in Figure 10. Representative classified output images from the HAM10000 dataset are shown in Figure 11. Representative classified output images from the ISIC 2019 dataset are shown in Figure 12.

**Figure 8:**
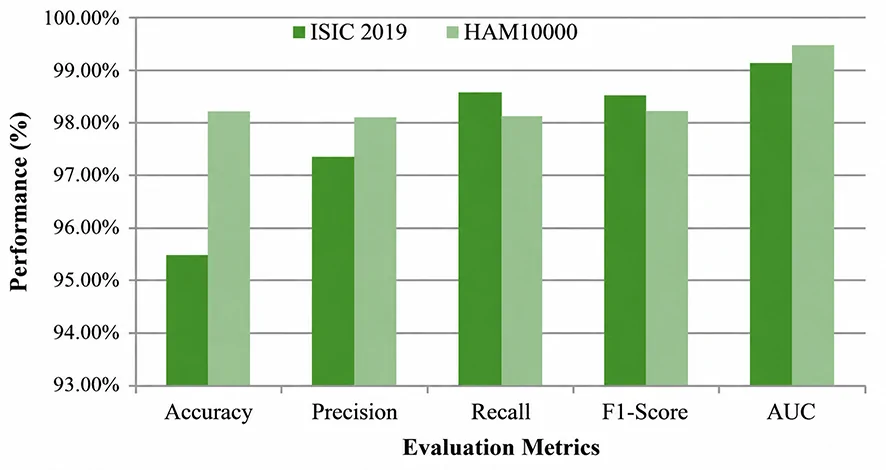
Quantitative comparison of classification performance across ISIC 2019 and HAM10000 datasets.

**Figure 9:**
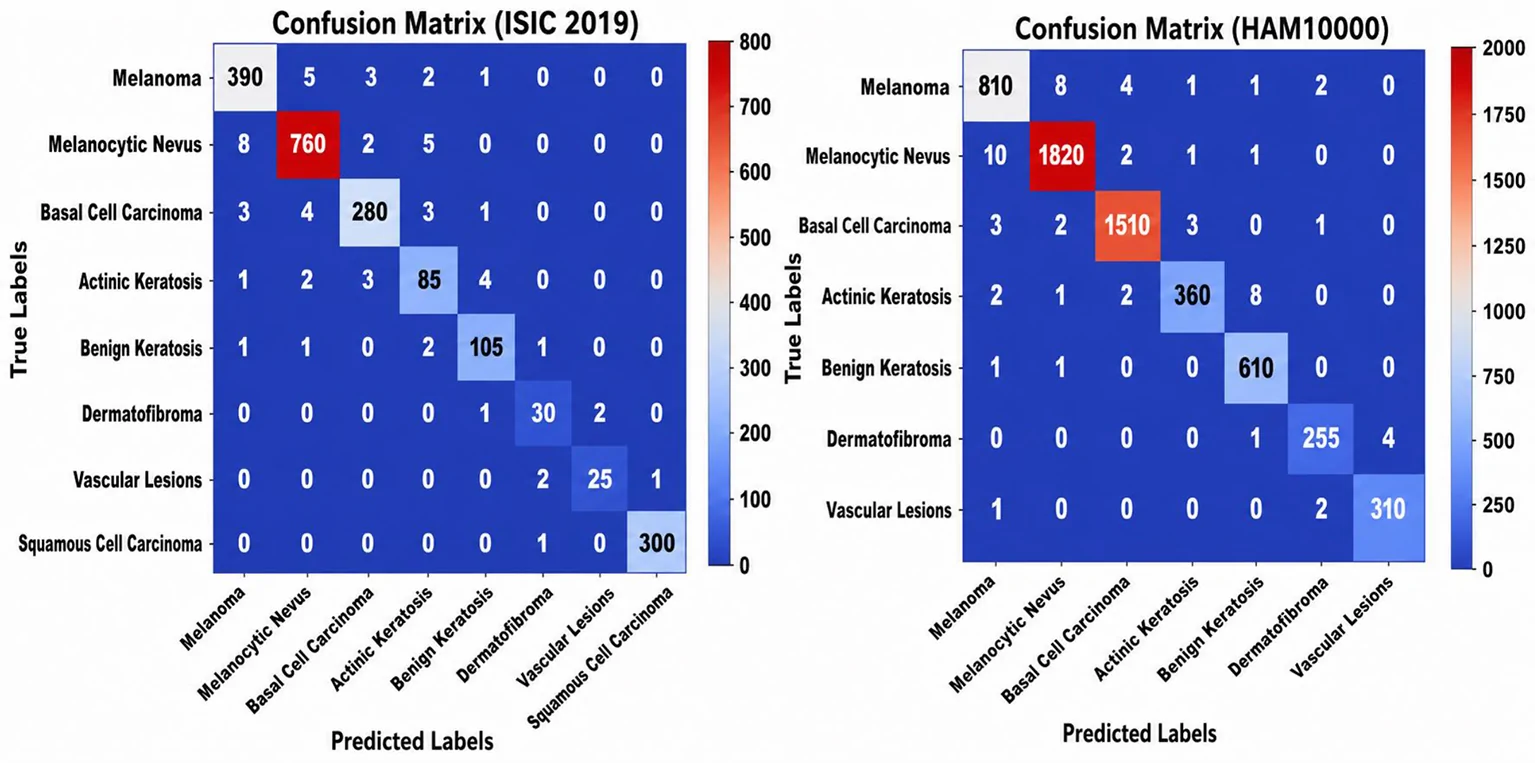
Confusion matrices for multi class classification on ISIC 2019 and HAM10000 datasets.

**Figure 10:**
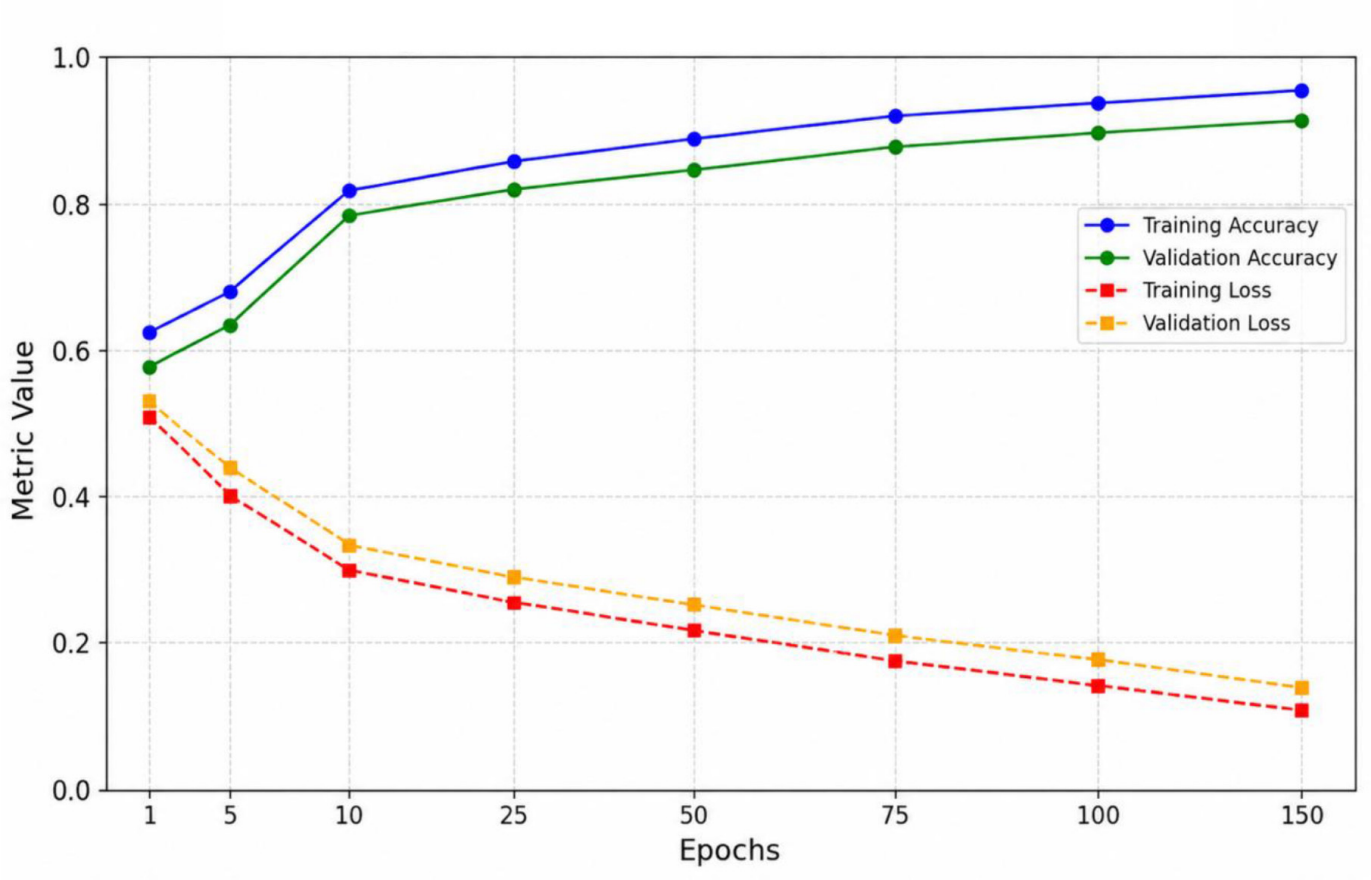
Training and validation accuracy and loss curves over 150 training epochs.

**Figure 11:**
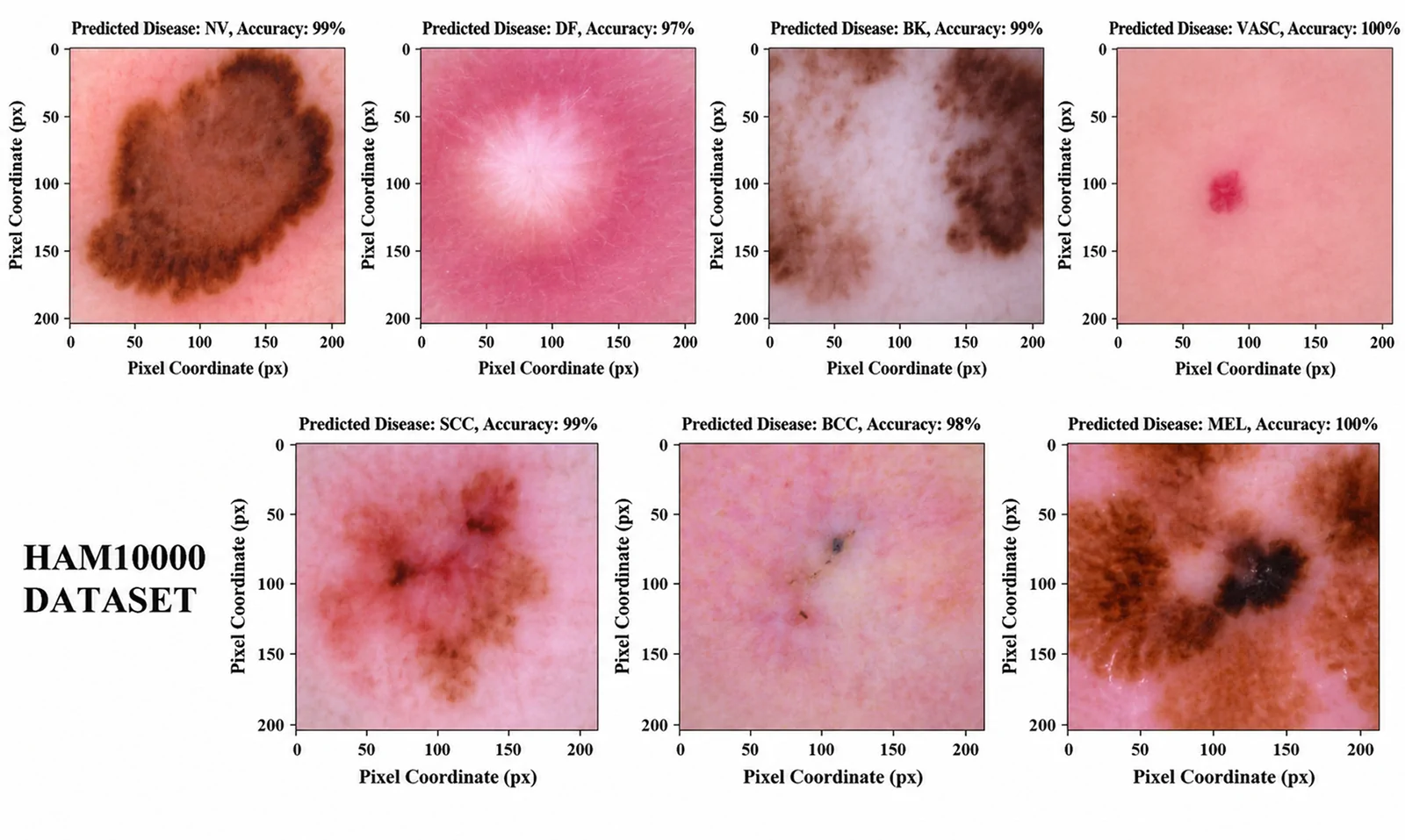
Sample classified output images from the HAM10000 dataset using the proposed framework.

**Figure 12:**
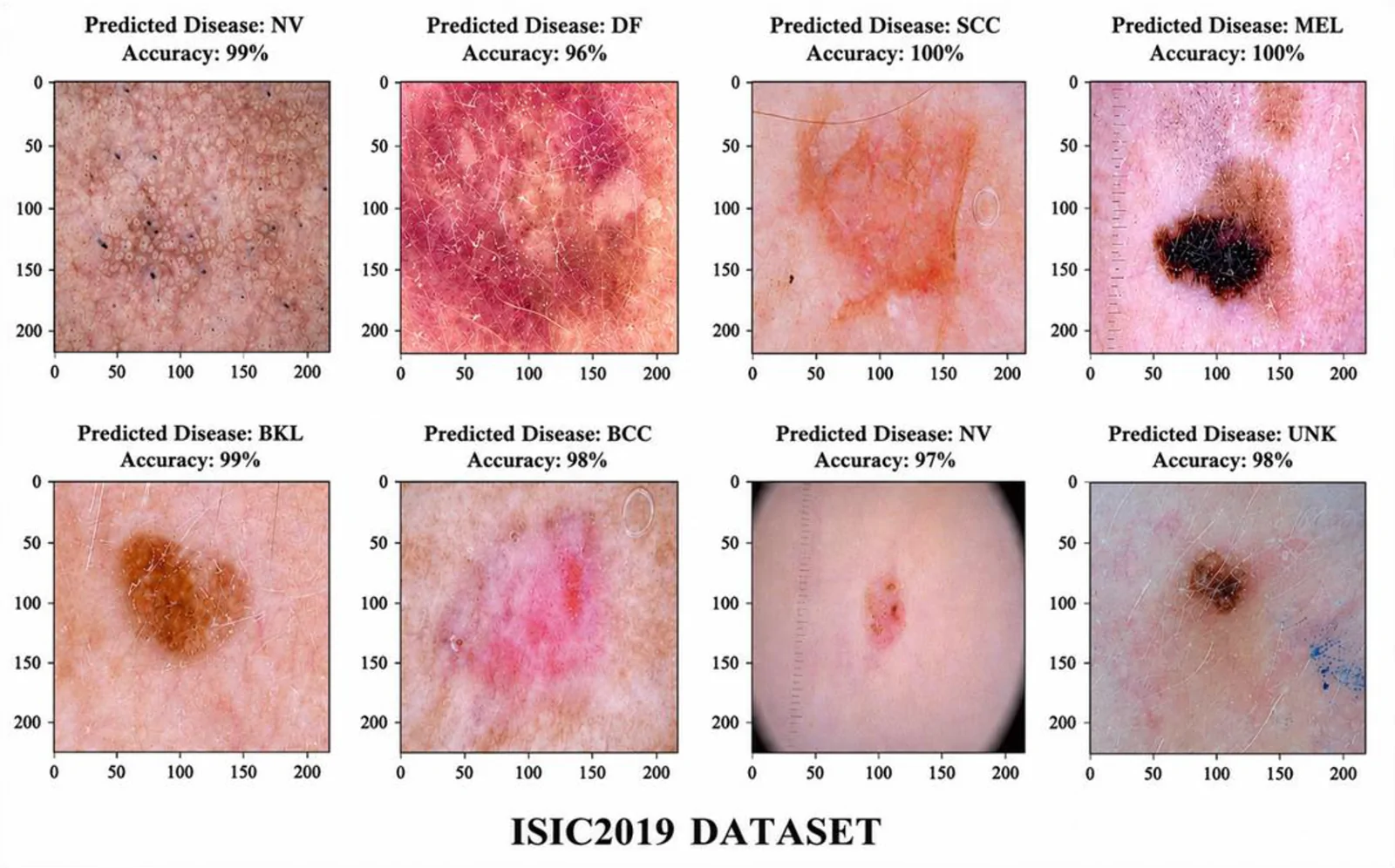
Sample classified output images from the ISIC 2019 dataset using the proposed framework.

The classification results indicate that optimization based feature selection contributes to improved discrimination between lesion types.

### 4.9 Ablation study

The individual contributions of each module in the proposed SkinNet-X architecture were assessed using a component wise ablation study. The ablation in Table 10 follows a progressive integration strategy and therefore evaluates the incremental contribution of the proposed modules rather than a fully factorial independent ablation. COA is introduced as the global exploration stage, and HWOA is subsequently added as the local refinement stage of the proposed sequential dual-stage optimization. Accordingly, the observed improvement after adding HWOA should be interpreted as the incremental benefit of local refinement on top of COA rather than evidence that HWOA independently outperforms COA.

**Table 10:** Component wise Ablation study of the proposed SkinNet-X framework.

Configuration	Dice (%)	IoU (%)	Classification accuracy (%)	Improvement
Baseline (Swin + EfficientNet)	91.84	85.92	95.21	Baseline
+ SAM refinement	92.76	87.08	95.86	Improved lesion boundary localization
+ CTCL	93.91	89.08	97.12	Better semantic consistency
+ COA feature optimization	94.21	89.46	97.58	Improved feature selection
+ HWOA refinement	94.53	89.63	97.91	Better local feature refinement
+ PolyLoss	94.81	90.13	98.18	Stable optimization
Full SkinNet-X	95.12	90.18	98.46	Best overall performance

SAM → better lesion boundaries; CTCL → better cooperation between segmentation and classification;

COA → improves feature exploration; HWOA → refines feature representation; PolyLoss → stabilizes optimization.

Each module of the proposed SkinNet-X framework contributes favorably to the overall performance, according to the component wise ablation analysis. Segmentation and classification performance are consistently improved by gradually integrating the SAM refinement module, Cross Task Consistency Learning (CTCL), training time dual stage feature optimization, and PolyLoss optimization. By specifically lowering semantic inconsistency between lesion location and illness detection, CTCL offers the largest performance boost among these elements. The higher performance of the whole SkinNet-X framework illustrates that the observed gains emerge from the complementing interplay of all proposed components rather than from any specific module alone. The baseline multitask architecture achieved a Dice coefficient of 91.84%, an IoU of 85.92%, and a classification accuracy of 95.21%. Progressive integration of the proposed components subsequently improved both segmentation and classification performance, with the complete SkinNet-X framework achieving 95.12% Dice, 90.18% IoU, and 98.46% classification accuracy. The component-wise ablation in Table 10 primarily evaluates the learning and optimization modules introduced after preprocessing. Accordingly, the table should not be interpreted as independently quantifying the contribution of CLAHE, hair-artifact removal, and U-Net denoising. These preprocessing operations were retained consistently across the reported configurations. A dedicated preprocessing ablation, including comparison with modern generative enhancement strategies, would provide additional insight and is considered a useful direction for future investigation. SAM and CRF perform different stages of refinement in SkinNet-X. SAM operates within the segmentation pathway to refine decoder-generated lesion boundaries, whereas CRF is applied only as a final post-processing operation to improve pixel-label consistency. Therefore, the SAM entry in Table 10 refers to the contribution of SAM within the segmentation branch; CRF is treated separately as post-processing and should not be interpreted as part of the SAM module itself.

### 4.10 Statistical validation

Every experiment was conducted three times under the same circumstances. The presented values indicate the mean and standard deviation. The proposed framework's stability and dependability are validated by the low deviation between runs.

Each experiment was repeated three times under the same experimental configuration using different random initializations. Table 11 therefore reports the mean and standard deviation across the repeated runs as an assessment of numerical stability. The relatively small variation between runs indicates consistent model convergence. Since the present repeated-run analysis was not designed as a paired hypothesis test against a predefined reference model, unsupported inferential p-values have been removed to avoid over interpretation of statistical significance.

**Table 11:** Statistical validation of the proposed SkinNet-X framework.

Metric	Mean (%)	Standard deviation
Dice	95.12	0.21
IoU	90.18	0.27
Classification accuracy	98.46	0.18
Precision	98.21	0.24
Recall	98.39	0.22
F1-score	98.30	0.20

### 4.11 Discussion

Through explicit cross task semantic interaction, the experimental findings show that the proposed SkinNet-X framework successfully enhances both skin lesion segmentation and illness categorization. Our framework introduces Cross Task Consistency Learning (CTCL) to explicitly regularize the semantic relationship between lesion localization and disease recognition throughout the optimization process, in contrast to traditional multitask learning approaches that mainly rely on shared feature extraction or attention based fusion. CTCL promotes the segmentation and classification branches to learn mutually complementing representations by consistently reducing semantic disagreement between the two learning objectives. This leads to better multitask cooperation and more stable optimization.

The segmentation findings on ISIC 2018 show that the proposed framework maintains robust lesion boundary delineation across lesions with irregular shapes, poor contrast, and diverse appearance. Effective modeling of both long range contextual information and local structural features is made possible by the incorporation of the Swin Transformer encoder, while boundary continuity is further enhanced by the lightweight SAM refinement module, which corrects coarse segmentation outputs. Consequently, Consequently, the predicted lesion masks closely mirror the manually annotated ground-truth masks on the ISIC 2018 test set. Lesion guided feature learning significantly enhances illness recognition performance, as shown by the classification findings. Background interference is greatly minimized and the network focuses mostly on clinically important lesion features since the segmentation branch generates lesion focused semantic representations for the classification branch. Additionally, by integrating global feature exploration with adaptive local refinement, the proposed training time dual stage feature optimization technique increases feature discriminability while improving optimization stability without raising inference complexity.

The significance of the proposed consistency regularization in multitask optimization is further confirmed by the CTCL sensitivity analysis. While extremely tiny weights fail to establish sufficient semantic interaction and excessively high weights significantly restrict task specific learning, moderate consistency weights consistently offer the optimum compromise between segmentation and classification performance. These findings suggest that explicit semantic consistency offers a useful method for enhancing cooperation amongst diverse prediction tasks while maintaining each one's capacity for discrimination.

Additionally, the component wise ablation analysis shows that each significant module enhances the framework as a whole. Lesion localization and illness classification are continuously improved by the progressive integration of the SAM refinement module, CTCL mechanism, training time dual stage feature optimization, and PolyLoss optimization. These results confirm that the complementing interplay of several learning modules within the proposed multitask framework, rather than a single architectural element, is responsible for the observed performance gains. In addition to predictive effectiveness, Our methodology displays good computational efficiency. According to the complexity study, SkinNet-X achieves rapid inference speed appropriate for near real time execution while maintaining a reasonable amount of trainable parameters and floating point operations. There is no additional computational cost during deployment since the COA-HWOA optimization approach only functions during training. This trait makes Our system more viable for computer aided dermatological diagnostics compared with more computationally expensive multitask designs. All things considered, the experimental results consistently show that explicit cross task semantic consistency is a useful tactic for enhancing multitask medical picture processing.

By jointly optimizing lesion segmentation and disease classification through semantic representation alignment, SkinNet-X achieves robust segmentation accuracy, reliable classification performance, stable convergence behavior, and efficient computational complexity across multiple benchmark datasets. These results support the effectiveness of The developed method while also providing a strong foundation for future multitask medical image analysis systems. From a clinical perspective, accurate lesion boundary delineation supports improved visual interpretation and facilitates reliable diagnostic assessment. By jointly optimizing segmentation and classification, the proposed framework provides complementary information that may assist dermatologists during lesion screening while reducing manual interpretation variability. Nevertheless, prospective clinical validation across diverse populations remains necessary before routine deployment.

In our previous work, we introduced **AttenUNeT-X**, an enhanced UNet based skin lesion segmentation framework that incorporated an iterative feedback mechanism, an Order Statistics Layer (OSL), and attention modules to improve lesion boundary delineation and contextual feature representation. The framework demonstrated robust segmentation performance across the ISIC 2018, ISIC 2017, and PH2 benchmark datasets, showing that progressive feature refinement can significantly enhance boundary localization and segmentation accuracy. Nevertheless, the proposed model was specifically designed for lesion segmentation and did not investigate the semantic interaction between lesion localization and disease classification. Consequently, segmentation and classification were optimized independently without explicit cross task knowledge transfer, motivating the development of the present SkinNet-X framework that jointly learns both tasks through Cross Task Consistency Learning (CTCL).

### 4.12 Limitation

Although SkinNet-X demonstrates strong performance on publicly available benchmark datasets, the present study has several limitations. First, the evaluation was conducted exclusively on publicly available dermoscopic datasets, and external clinical validation across independent institutions was not performed. Second, the proposed framework involves higher computational complexity than conventional single-task networks because of its integrated multitask architecture and multiple processing components. Third, the proposed CTCL mechanism was evaluated specifically for skin lesion segmentation and classification, and its applicability to other medical imaging tasks remains to be investigated. In addition, k-fold cross-validation was not performed because repeated retraining of the complete multi-stage framework would impose substantial computational requirements. Although repeated experimental runs were conducted to assess model stability, future studies will investigate cross-institutional validation, k-fold and multi-partition evaluation, lightweight deployment, and the extension of CTCL to other medical imaging and multimodal applications. Furthermore, quantitative segmentation evaluation was restricted to ISIC 2018 because ISIC 2019 and HAM10000 were used as classification datasets without corresponding pixel-level segmentation annotations in the present study.

## 5 Conclusion

This paper presented SkinNet-X, a unified multitask deep learning framework for simultaneous skin lesion segmentation and classification. Dissimilarly conventional multitask learning approaches that primarily rely on implicit feature sharing, The developed method introduces a novel Cross Task Consistency Learning (CTCL) mechanism to explicitly enforce semantic consistency between lesion localization and disease recognition during joint optimization. By integrating adaptive preprocessing, Swin Transformer based segmentation, SAM guided boundary refinement, CTCL, COA-HWOA feature optimization, and PolyLoss driven optimization, the proposed framework effectively improves both segmentation quality and classification performance within a single end-to-end architecture. Comprehensive experimental evaluation using ISIC 2018 for lesion segmentation and ISIC 2019 and HAM10000 for disease classification demonstrated the effectiveness and robustness of the proposed framework across complementary dermatological learning tasks.

### 5.1 Failure case analysis

The proposed framework still exhibits limitations for dermoscopic images with extremely low contrast, substantial illumination variation, thick hair occlusion, and highly irregular lesion boundaries. The present benchmark datasets do not provide standardized test-set annotations categorizing images according to these artifact conditions; therefore, the current failure-case assessment is qualitative rather than a controlled artifact-stratified quantitative analysis. Representative errors indicate that segmentation inaccuracies under severe image degradation may propagate to the classification pathway. Future work will therefore include artifact-specific evaluation using explicitly annotated low-contrast, illumination-variation, hair-occlusion, and irregular-boundary subsets.

Figure 13 Representative ISIC 2018 segmentation failure cases of SkinNet-X under challenging dermoscopic conditions. The examples illustrate segmentation errors associated with low lesion-to-skin contrast, dense hair occlusion, illumination variation, and highly irregular lesion boundaries. Ground-truth and predicted masks are shown for visual comparison. These cases demonstrate that severe image artifacts and ambiguous boundary characteristics can still affect lesion localization and may subsequently influence classification.

**Figure 13:**
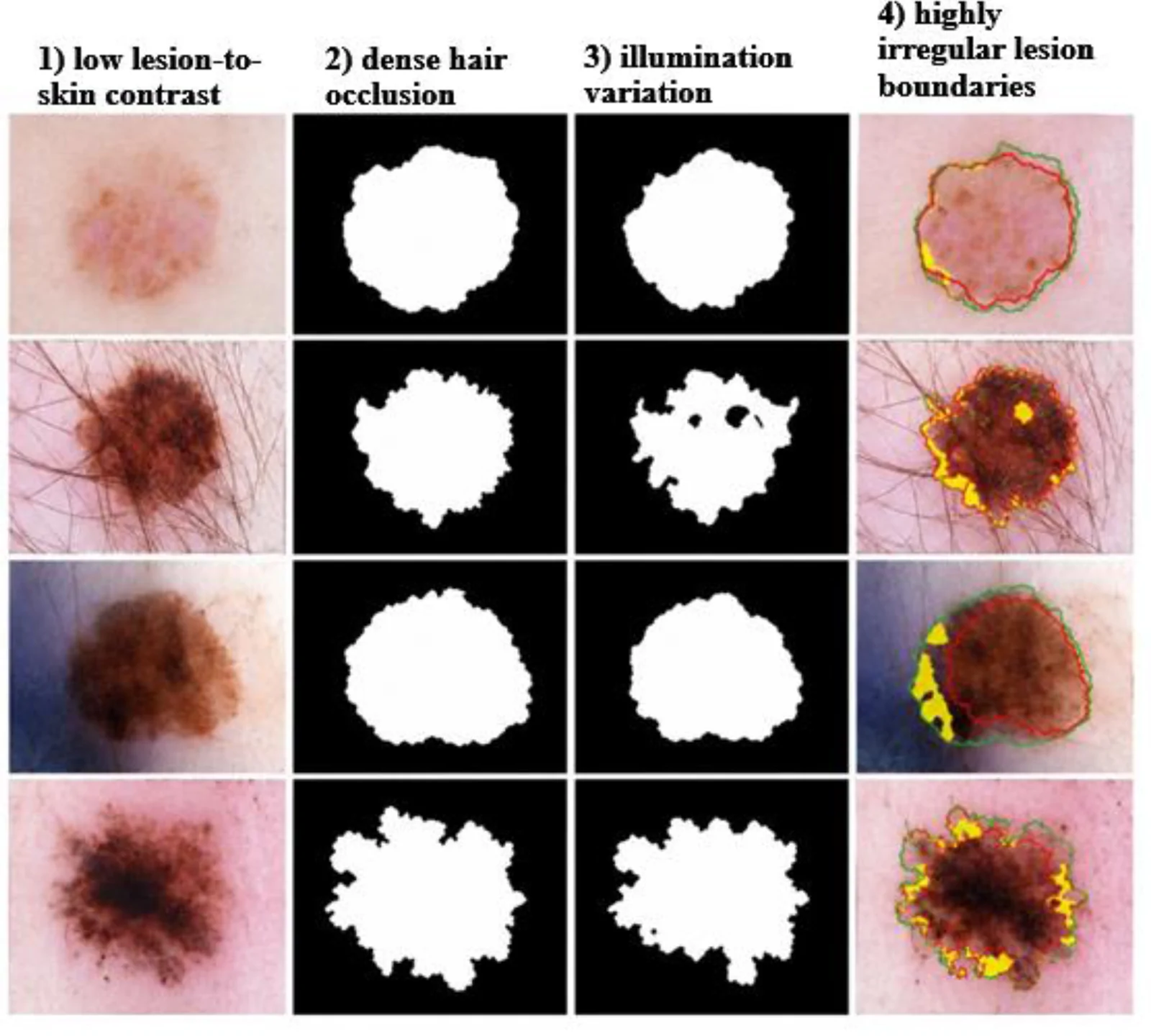
Representative ISIC 2018 segmentation failure cases of SkinNet-X under challenging dermoscopic conditions.

## Data Availability

The original contributions presented in the study are included in the article/supplementary material, further inquiries can be directed to the corresponding author.
